# A glycine at position 105 leads to clavulanic acid and avibactam resistance in class A β-lactamases

**DOI:** 10.1016/j.jbc.2025.110347

**Published:** 2025-06-06

**Authors:** Marko Radojković, Aleksandra Chikunova, Saar F. Koene, Monika Timmer, Sivanandam V. Natarajan, Aimee L. Boyle, Marcellus Ubbink

**Affiliations:** 1 Department of Macromolecular Biochemistry, Leiden Institute of Chemistry, Leiden University, Leiden, The Netherlands; 2 The Netherlands Cancer Institute, Amsterdam, The Netherlands; 3 School of Chemistry, University of Bristol, Bristol, United Kingdom

**Keywords:** inhibitor resistance, beta-lactamase, clavulanic acid, avibactam, deep sequencing

## Abstract

β-Lactamase enzymes exhibit extraordinary adaptive potential, thus rendering many β-lactam drugs ineffective. The residue at Ambler position 105, also known as the gatekeeper residue, plays an important role in substrate recognition, but its implication in inhibition mechanisms remains understudied and obscure. To inspect the relationship between inhibitor-resistant phenotypes and residues at this position, we performed site-saturation mutagenesis and extensive fitness profiling of five distinct class A β-lactamases using deep sequencing. We found that inhibitor resistance is readily detectable, with variants harboring Gly or Arg being the least susceptible to inhibitors. Mutation of Ile105 to Arg in the β-lactamase BlaC simultaneously enhances activity for carbenicillin and the ability to evade clavulanic acid inhibition. The Y105G substitution in two clinically important enzymes, CTX-M-14 and TEM-1, confers greatly reduced *in vitro* sensitivity to avibactam, which we attribute to elevated conformational flexibility of the inhibitor within the active site. The findings presented in this study underpin the gatekeeper residue as a possible mutational hotspot and might aid the design of novel β-lactamase inhibitors.

Antibiotic resistance mediated by β-lactamase-producing bacteria has become one of the major threats to global health. This phenomenon has substantially reduced the efficacy of β-lactam drugs, which are the most commonly prescribed antibiotics worldwide (1). β-Lactamases (EC.3.5.2.6) are a group of enzymes that can hydrolyze the amide bond of the β-lactam ring, thus making the antibiotics inactive. Based on sequence homology, these enzymes can be divided into four classes (A-D) (2), of which classes A, C, and D have an active-site serine (serine β-lactamases, SBLs) and exploit catalysis with a covalent intermediate, whereas class B enzymes harbor one or two metal ion(s) in the active site (metallo-β-lactamases, MBLs). Since the emergence of bacterial resistance, class A remains the most widespread and extensively studied (3). Over the past decades, β-lactam molecules have undergone various optimizations to counteract the spread of bacterial SBLs (4, 5). Perhaps one of the most promising strategies was the implementation of β-lactam/β-lactamase inhibitor combinations. However, continued selection pressure exerted by these drugs led to the emergence of inhibitor-resistant variants (6, 7, 8, 9, 10), illustrating the capacity of these enzymes for evolutionary adaptation.

The interactions between the β-lactamase enzyme and ligand molecules rely on the intricate network of active-site residues. Every amino acid substitution has the potential to induce a subtle reorganization of the active site, which can drastically modify the activity profile and affect inhibitor binding. One such residue, at position 105 (standard Ambler numbering (2)), is suggested to play an important role in substrate discrimination of class A enzymes (11, 12, 13). Due to its location at the entrance of the active site, this residue has also been named “gatekeeper” (14). In most class A β-lactamases, this position is occupied by an amino acid with an aromatic side chain, including His. It was previously suggested that the strong aromatic bias stems from stabilizing interactions between the side chain of 105 and different substrate moieties, either through van der Waals and hydrophobic interactions with a thiazolidine ring (15) or stacking interactions with a benzyl or thienyl group (16, 17). Interestingly, BlaC, the β-lactamase from *Mycobacterium tuberculosis*, possesses Ile at this position, sparking the question about the evolutionary preference for this amino acid. We recently demonstrated that the majority of the substitutions at position 105 in BlaC led to increased bacterial fitness against a combination of clavulanic acid and carbenicillin, with surprisingly, Arg105 being the fittest variant (18). A similar trend was observed in KPC-2 β-lactamase, where more than half of the examined variants exhibited a 2- to 4-fold increase in MIC against clavulanic acid and ampicillin (13). Thus, it has been proposed that residue 105 acts as a determinant of susceptibility to mechanism-based inhibitors, by forming a hydrophobic wall that allows for the protonation of clavulanic acid during the chemical reactions that occur upon ring opening and causes its inhibitory nature (11). Although the relationship between this residue and β-lactam-based inhibitors has been relatively well described, the potential to evade inhibition by non-β-lactam inhibitors, such as avibactam, has never been evaluated in class A enzymes. Unlike clavulanic acid, avibactam is a reversible covalent inhibitor and does not display secondary chemistry upon binding.

With this in mind, we set out to explore substrate and inhibitor discrimination of residue 105 in BlaC. To seek general trends within the class A enzymes, we also included four additional β-lactamases. Site-saturation mutagenesis and screening combined with deep sequencing were performed against a panel of β-lactam antibiotics and inhibitors. Inhibitor-resistant phenotypes were identified for each enzyme, with a clear preference for Gly at this position. Besides reduced susceptibility to clavulanic acid, BlaC I105R and TEM-1 Y105G enzymes showed a 4- and 16-fold increase in bacterial resistance to avibactam, suggesting a common resistance mechanism. Structural investigations of BlaC I105R reveal a notably enlarged active site and possibly increased dynamics of the gatekeeper residue upon binding of the inhibitors. Our results provide an indication that inhibitor-resistant variants at this position might arise in the future.

## Results

### Substrate and inhibitor susceptibility of various class A β-lactamases

**Figure 1:**
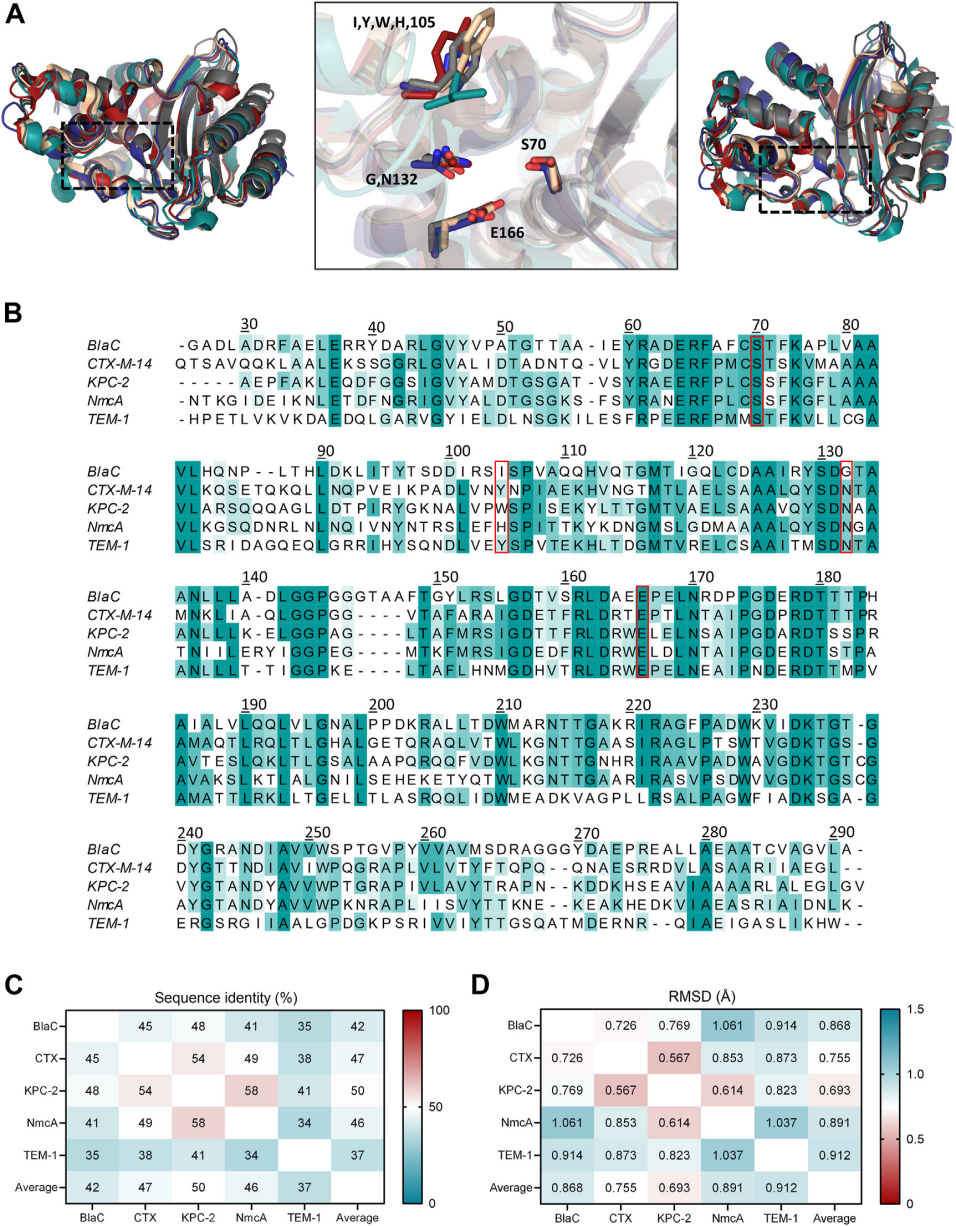
**Structural and sequence alignment of five class A β-lactamases.***A*, 3D-alignment of enzyme structures with PDB entries 2GDN (54) (BlaC, *cyan*), 1YLT (55) (CTX-M-14, *red*), 2OV5 (56) (KPC-2, *wheat*), 1BUE (57) (NmcA, *blue*) and 1BTL (58) (TEM-1, *gray*) generated by PyMoL. Aligned structures are shown from two perspectives: *left-frontview*, *right – topview*. The configuration of the active site is depicted in the *center*, with side chains of residues 70, 105, 132, and 166 shown as sticks. *B*, sequence alignment of the β-lactamases generated by ClustalOmega (65) and visualized using Jalview (66). Residues are annotated according to the Ambler numbering and correspond to residue numbers 41-307 of BlaC (Uniprot entry P9WKD3-1), 29 to 291 of CTX-M-14 (Q9L5C7), 30 to 293 of KPC-2 (Q9F663), 28 to 292 of NmcA (P52663), and 24 to 286 of TEM-1 (P62593). The *green* shading indicates the degree of sequence identity. Active site residues shown in panel (*A*) are highlighted with *redboxes*. *C*, sequence identity as determined by ClustalOmega and plotted as a heatmap. *D*, RMSD values generated after alignment in PyMOL and plotted as a heatmap.

To evaluate general trends of substrate and inhibitor discrimination of wild-type enzymes, besides BlaC, we chose four other representative class A enzymes: CTX-M-14, KPC-2, NmcA, and TEM-1 (Fig. 1). The CTX-M and TEM families of enzymes are the most frequently found extended-spectrum β-lactamases (ESBLs) in Gram-negative bacteria and display strong hydrolytic activity toward penicillin β-lactams. KPC-2 and NmcA belong to a carbapenemase group of class A β-lactamases, enzymes that can efficiently break down carbapenem antibiotics. Although the average sequence identity between selected β-lactamases is relatively low (<50%, Fig. 1*C*), the 3D structures are remarkably similar (Fig. 1*A*), with an average RMSD of Cα atoms of 0.82 Å (Fig. 1*D*). Out of all enzymes, TEM-1 shares the least sequence analogy and structural resemblance with other class A orthologs (Fig. 1, *C* and *D*).

**Figure 2:**
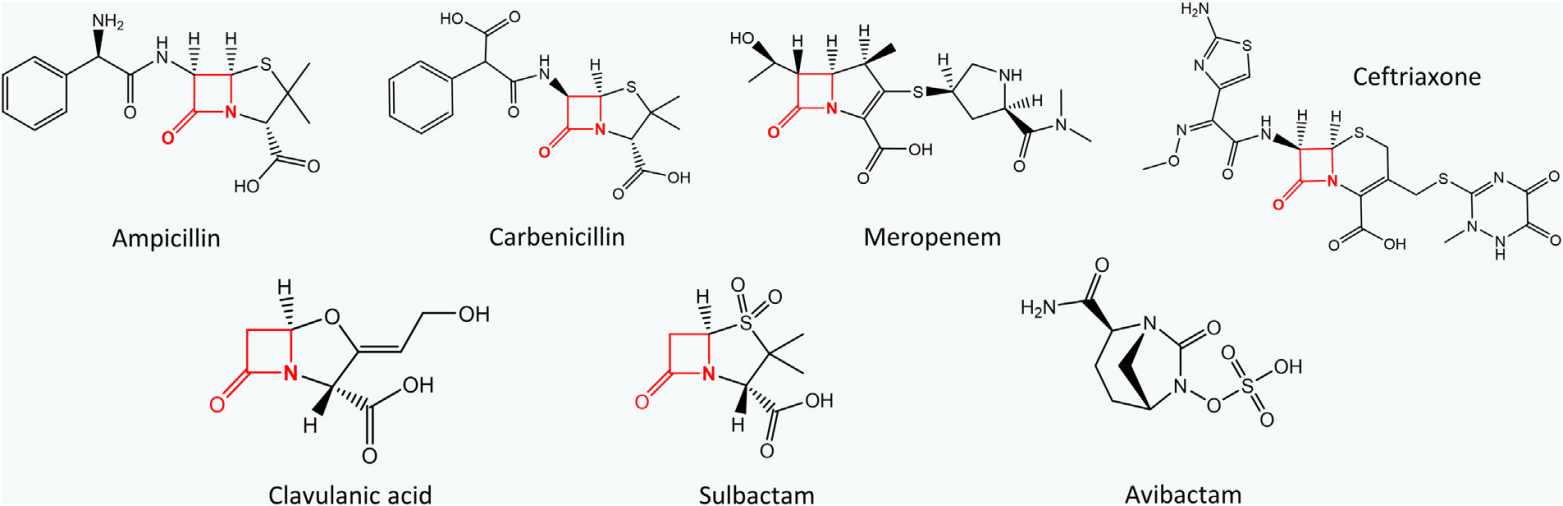
**Structures of β-lactam antibiotics and inhibitors used in susceptibility testing and fitness experiments.** The β-lactam ring in each molecule is highlighted in *red*. *Top* – β-lactam antibiotics; *bottom* – β-lactamase inhibitors.

The genes coding for the soluble parts of these enzymes were cloned into a pUK21 vector, preceded by a signal peptide for translocation to the bacterial periplasm, as previously described (18). BlaC, CTX-M-14, and KPC-2 enzymes were translocated *via* the Tat system (twin-arginine translocation pathway), whereas TEM-1 and NmcA enzymes were translocated via the Sec system (secretion pathway). Vectors carrying genes of wild-type variants were introduced into *Escherichia coli* cells, and bacterial susceptibility was assessed against a panel of β-lactam antibiotics and inhibitors (Fig. 2) by determining the minimum inhibitory concentration (MIC). As expected, CTX-M-14 and TEM-1 displayed high MIC values for both penicillin antibiotics, ampicillin and carbenicillin (Table 1). Likewise, KPC-2 and NmcA β-lactamases showed higher resistance against the carbapenem meropenem than the rest. Apart from CTX-M-14, all β-lactamases were highly sensitive to ceftriaxone. Interestingly, the presence of the conserved SDN motif (Ambler positions 130–132) proved to be an important determinant of inhibitor discrimination. All β-lactamases carrying this sequence were highly susceptible to avibactam and considerably less to the clavulanic acid and sulbactam. In contrast, BlaC, which instead of Asn harbors Gly at position 132, was markedly more resistant to avibactam, yet more susceptible to the other two inhibitors, as previously noted from *in vitro* characterization (19).

**Table 1:** MIC values (**μ**g/ml) for various substrates and inhibitors of five selected **β**-lactamases in *E. coli*

**β**-lactamase	AMP	CAR	MEM	CEF	CA^a^	SUL^a^	AVI^a^
pUK21^b^	10	10	0.05	0.1	/	/	/
BlaC (Tat^c^)	50	800	0.1	0.2	0.5	4	16
CTX-M-14 (Tat^c^)	1600	16,000	0.1	1600	1	32	0.5
KPC-2 (Tat^c^)	200	400	0.4	0.8	1	16	0.5
NmcA (pelB/Sec^c^)	400	200	1.6	0.2	2	32	0.5
TEM-1 (Sec^c^)	12,800	32,000	0.05	0.1	2	32	1

AMP, ampicillin; CAR, carbenicillin; MEM, meropenem; CEF, ceftriaxone; CA, clavulanic acid; SUL, sulbactam; AVI, avibactam.

a In combination with 100 μg/ml (BlaC, CTX-M-14, KPC-2, and TEM-1) and 25 μg/ml carbenicillin (NmcA).

b pUK21 vector without the β-lactamase gene.

c Translocation system used.

### Deep sequencing of gatekeeper residue libraries

Site-saturation libraries of residue 105 in all five β-lactamases were constructed using mutagenic primers carrying NNS or NNK degeneracy. These single-mutant libraries were afterward introduced into *E. coli* cells for the determination of fitness under the selection pressure of all compounds used in susceptibility testing (Fig. 2). Inoculated liquid cultures without (no selection) or with antibiotic (and inhibitor) at previously determined MIC values (Table 1) were incubated at 37 °C.

Deep sequencing was performed using Illumina 300 bp paired-end sequencing (PE), and counts for each variant under each condition were obtained. Reads were then quality filtered and the relative fitness effect (*F*_*i*_) of each variant was determined as described previously (18, 20), where *F*_*i*_ is calculated as the logarithm of the ratio of the allele counts in the populations obtained under selection (*N*_i_^*sel*^) and without selection (*N*_i_^*unsel*^), relative to the wild-type allele, as shown in Equation 1:

(1) $$F_{i}=\log_{10}\left[\frac{N_{i}^{sel}}{N_{i}^{unsel}}\right]-\log_{10}\left[\frac{N_{i}^{wt,sel}}{N_{i}^{wt,unsel}}\right]$$

Library variants showing beneficial or deleterious fitness relative to the wild-type have positive or negative *F*_i_ values, respectively. The significance of *F*_i_ values was determined based on errors derived from biological replicates of each variant and wild-type enzymes.

**Figure 3:**
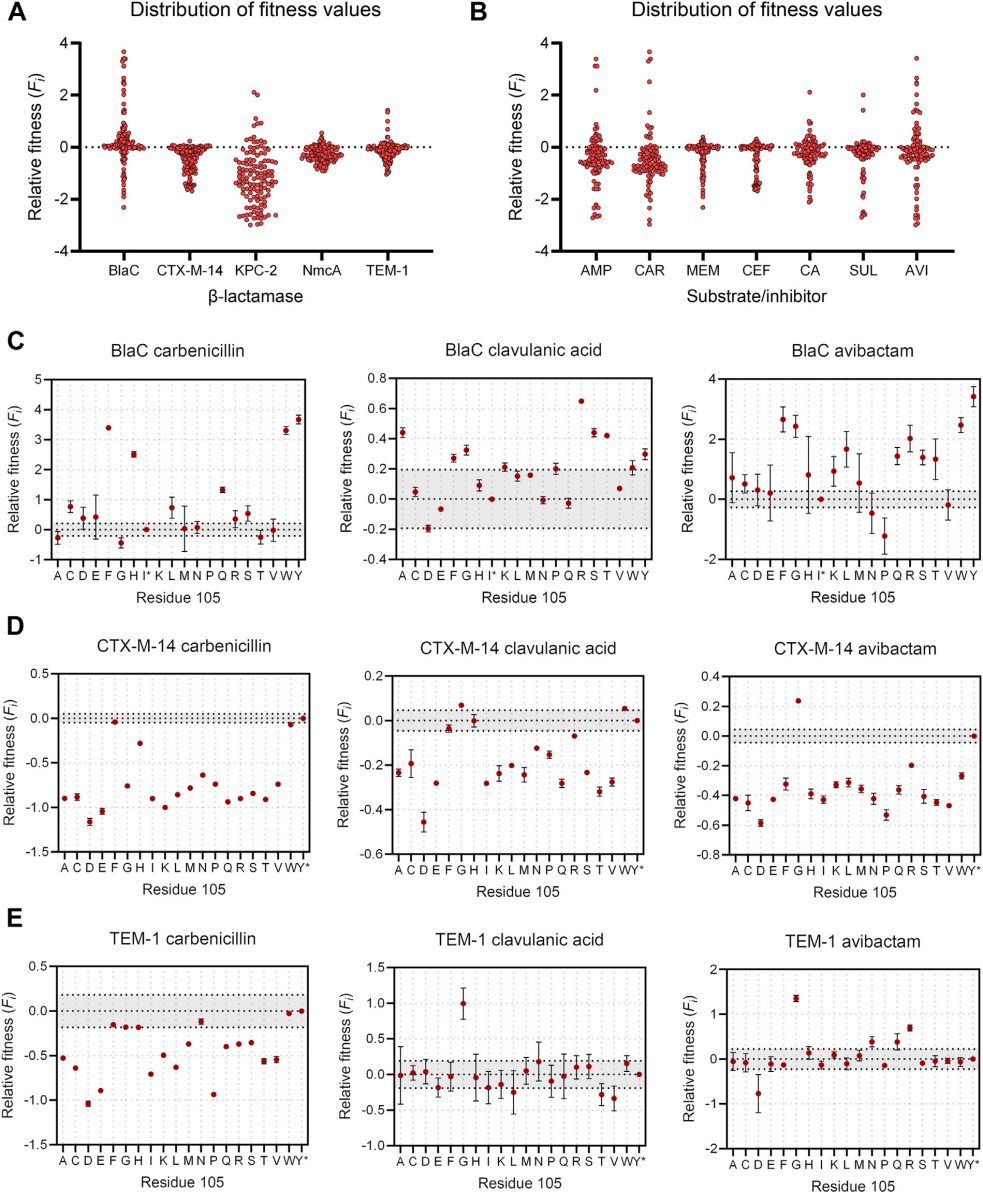
**Deep sequencing results.** Fitness values relative to the wild-type are calculated according to Equation 1. (a-b) Distribution of all fitness values. Fitness values are plotted per β-lactamase (*A*) or substrate/inhibitor (*B*). *C*–*E*, fitness values for carbenicillin, clavulanic acid, and avibactam, plotted for each amino acid variant at position 105; (*C*) BlaC; (*D*) CTX-M-14; (*E*) TEM-1. *Gray* highlight around zero denotes variability in absolute wild-type fitness (Δ^abs^*F*_*i*_), which was used to determine the significance of relative fitness values (see Experimental procedures). Error bars represent the average error or standard deviation of duplicate or triplicate datasets, respectively.

The distribution of fitness values of all variants is shown in Figure 3, where the fitness is plotted per β-lactamase (Fig. 3*A*) or antibiotic (and inhibitor) (Fig. 3*B*). The vast majority of the substitutions lead to a negative fitness, regardless of the enzyme or substrate tested. To our surprise, BlaC had the highest number of beneficial mutations and is the only enzyme that had more positive than negative fitness values (Supporting Data). Most of the library variants of other enzymes performed worse than the wild types and had a similar distribution profile, with values slightly to moderately negative.

The exception is KPC-2 β-lactamase, for which the negative fitness values are dispersed over a wider range and, on average, more negative than those of the other β-lactamases (Fig. 3*A*). By looking at the distributions per type of β-lactam antibiotic, the type of residue at position 105 appears to strongly influence the resistance toward penicillin antibiotics and weakly influence ceftriaxone or meropenem resistance. More variants displayed deleterious rather than beneficial fitness against the latter two substrates. Interestingly, among inhibitors, we found that selection under avibactam yielded the most variants with positive fitness values, followed by clavulanic acid.

We then closely looked at the variants that showed increased fitness against these two inhibitors. Following our previous observation, BlaC had the most variants with positive fitness, of which 7 performed significantly better than the wild-type for clavulanic acid and 9 for avibactam (Fig. 3*C*). In many cases, these variants also display strong fitness in the presence of carbenicillin only, which was the antibiotic used in combination with the inhibitors. This was not the case for the variants I105R and I105G, which were not significantly better against carbenicillin but showed a beneficial phenotype against both inhibitors. Also in other β-lactamases, and particularly TEM-1, replacement of the wild-type residue with Gly led to significantly increased fitness against clavulanic acid and avibactam (Figs. 3, *D* and *E*, S1). To examine whether increased fitness translates to elevated MIC values, we prepared Gly and Arg mutants of the gatekeeper residue for BlaC, CTX-M-14, and TEM-1 β-lactamases and determined their corresponding MIC values (Table 2).

**Table 2:** MIC values (**μ**g/ml) for carbenicillin, clavulanic acid, and avibactam of BlaC, CTX-M-14, and TEM-1 **β**-lactamases and their corresponding mutants at position 105

**β**-lactamase	Carbenicillin	Clavulanic acid^a^	Avibactam^a^
pUK21^b^	10	/	/
BlaC WT	800	0.5	16
BlaC I105R	1600	4	64
BlaC I105G	800	1	32
CTX-M-14 WT	16,000	1	0.5
CTX-M-14 Y105R	2000	1	1
CTX-M-14 Y105G	1000	1	2
TEM-1 WT	32,000	2	1
TEM-1 Y105R	4000	2	4
TEM-1 Y105G	16,000	32	16

a In combination with 100 μg/ml carbenicillin.

b pUK21 vector without β-lactamase gene.

Generally, we found a good correlation between MIC and fitness phenotypes for all three tested β-lactamases. The beneficial fitness of BlaC I105R correlates with an 8- and 4-fold increase in MIC values for clavulanic acid and avibactam, respectively. Likewise, the Y105G variant of CTX-M-14 raised the MIC value for avibactam by a factor of 4, although the resistance against carbenicillin dropped dramatically (16-fold decrease). Strikingly, the replacement of Tyr with Gly in TEM-1 led to a 16-fold increase in MIC for both inhibitors, without losing much of the resistance against carbenicillin (only a 2-fold decrease in comparison to the wild type).

### Inhibitor resistance and activity trade-off

**Figure 4:**
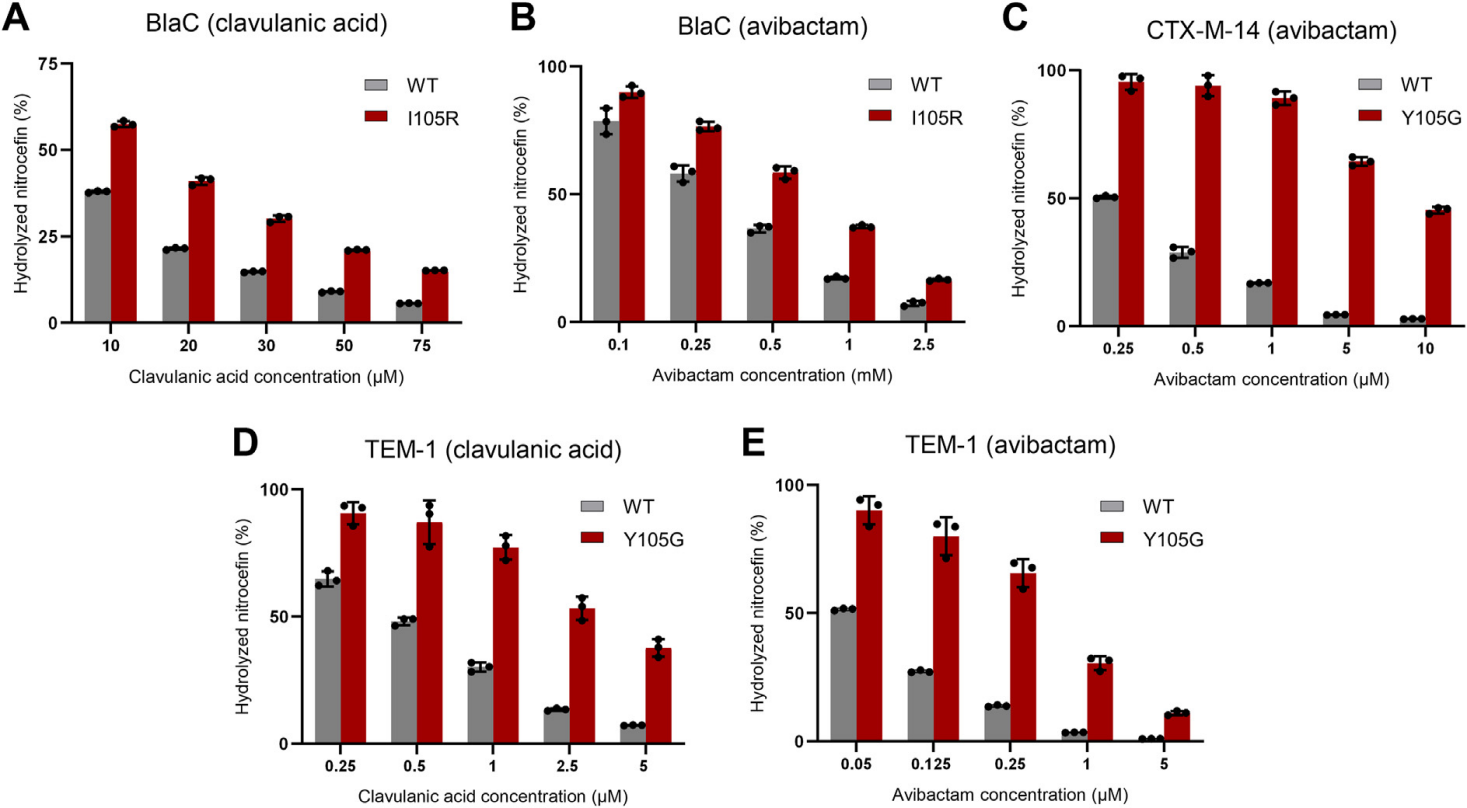
**Inhibitor susceptibility of BlaC, CTX-M-14, and TEM-1 wild-type enzymes, together with their corresponding 105 mutants.***A* and *B:* BlaC; (*C*) CTX-M-14; (*D* and *E*) TEM-1. The amount of hydrolyzed nitrocefin after 10 min in the presence of different inhibitor concentrations is expressed relative to the control (no inhibitor). The enzyme concentration was 2 nM (BlaC), 0.2 nM (CTX-M-14), or 0.2 and 0.25 nM (TEM-1). All measurements were done in 100 mM NaPi buffer, pH 6.4, at 25 °C. The error bars represent one standard deviation of the triplicate measurement. *Black circles* represent individual data points.

Next, we set out to elucidate biochemical determinants underlying bacterial resistance phenotypes of variants that were least susceptible to clavulanic acid and avibactam. To that end, we produced and purified BlaC I105R, CTX-M-14 Y105G, and TEM-1 Y105G, together with the wild-type enzymes. Steady-state kinetic parameters were subsequently determined for three substrates: nitrocefin, ampicillin, and carbenicillin (Table 3). *In vitro* susceptibility to the inhibitors was assessed by monitoring nitrocefin hydrolysis in the presence of various inhibitor concentrations for 10 min (Fig. 4).

**Table 3:** Steady-state kinetic parameters determined for BlaC, CTX-M-14, TEM-1, and their corresponding residue 105 mutants

	Nitrocefin			*k*_cat_*/K*_M_^app^ relative to the wild-type
	*K*_M_^app^ (μM)	*k*_cat_ (s^−1^)	*k*_cat_/*K*_M_^app^ (μM^−1^ s^−1^)	
BlaC WT (67)	131 ± 2	86 ± 2	0.65 ± 0.01	1
BlaC I105R	200 ± 20	24 ± 2	0.12 ± 0.01	0.18
CTX-M-14 WT	79 ± 1	576 ± 5	7.31 ± 0.08	1
CTX-M-14 Y105G	700 ± 172	963 ± 117	1.4 ± 0.4	0.19
TEM-1 WT	54 ± 9	933 ± 33	17 ± 3	1
TEM-1 Y105G	721 ± 186	453 ± 80	0.6 ± 0.2	0.04


	Ampicillin			*k*_cat_*/K*_M_^app^ relative to the wild-type
	*K*_M_^app^ (μM)	*k*_cat_ (s^−1^)	*k*_cat_/*K*_M_^app^ (μM^−1^ s^−1^)	
BlaC WT	68 ± 3	14 ± 2	0.21 ± 0.01	1
BlaC I105R	53 ± 1	3.4 ± 0.1	0.065 ± 0.001	0.31
CTX-M-14 WT	76 ± 2	103 ± 1	1.36 ± 0.08	1
CTX-M-14 Y105G	297 ± 10	48 ± 2	0.16 ± 0.01	0.11
TEM-1 WT	72 ± 1	2359 ± 16	33.0 ± 0.7	1
TEM-1 Y105G	107 ± 5	601 ± 9	5.6 ± 0.3	0.17


	Carbenicillin			*k*_cat_*/K*_M_^app^ relative to the wild-type
	*K*_M_^app^ (μM)	*k*_cat_ (s^−1^)	*k*_cat_/*K*_M_^app^ (μM^−1^ s^−1^)	
BlaC WT	5.5 ± 0.7	0.51 ± 00.2	0.09 ± 0.01	1
BlaC I105R	1.2 ± 0.4	0.27 ± 0.01	0.24 ± 0.07	2.7
CTX-M-14 WT	20 ± 2	31 ± 2	1.55 ± 0.18	1
CTX-M-14 Y105G	228 ± 61	11 ± 2	0.05 ± 0.02	0.03
TEM-1 WT	10.0 ± 0.7	92 ± 2	9.15 ± 0.66	1
TEM-1 Y105G	43 ± 5	59.0 ± 0.3	1.38 ± 0.16	0.15

All measurements were done in phosphate buffer (100 NaPi, pH 6.4) at 25 °C. Errors represent one standard deviation of the mean of triplicate measurements. The *K*_M_ is indicated as apparent because regular Michaelis-Menten kinetics do not apply to the two-step hydrolysis reaction (21).

In line with the observed MIC value, BlaC I105R showed improved activity (*k*_cat_/*K*_M_^app^) for carbenicillin (2.7-fold increase) but impaired hydrolysis of nitrocefin and ampicillin (4- and 3-fold reduction, respectively). The increased *k*_cat_/*K*_M_ ratio for carbenicillin likely stems from improved substrate binding, which is reflected in the lower *K*_M_ value than that of the wild-type. We note that generally very low *K*_M_ values are presumably due to the slow deacylation rate of carbenicillin, rather than high affinity (21). BlaC I105R is less sensitive to clavulanic acid and avibactam inhibition, although the effect is more prominent for the former inhibitor (Fig. 4, *A* and *B*). Both CTX-M-14 and TEM-1 Y105G variants are less active than their wild-type counterparts for all three tested substrates (Table 3). The *k*_cat_/*K*_M_ ratio of TEM-1 Y105G for carbenicillin is 6.6 times lower than that of the wild type, which is surprising, given that the MIC value for the same antibiotic only dropped 2 times (Table 2). Furthermore, TEM-1 Y105G showed a similar resistance profile to both clavulanic acid and avibactam (Fig. 4, *C* and *D*), albeit in comparison to the wild-type enzyme, the increased resistance seems to be more pronounced for the latter inhibitor (Fig. 4*D*). CTX-M-14 Y105G exhibited remarkably reduced sensitivity to avibactam (Fig. 4*B*), retaining almost 50% activity even at the highest inhibitor concentration tested.

The inhibition of BlaC I105R and TEM-1 Y105G by clavulanic acid was studied in more detail, using a reactivation model (22, 23), in which conversion of covalently bound clavulanic acid adduct EI∗ into a product P occurs with a rate constant *k*_*3*_, as shown in Equation 2:

(2) $$E+I\;\underset{k_{-1}}{\overset{k_{1}}{⇄}}\;\text{EI}\;\overset{k_{2}}{\to }\;EI^{*}\overset{k_{3}}{\to }E+P$$

**Figure 5:**
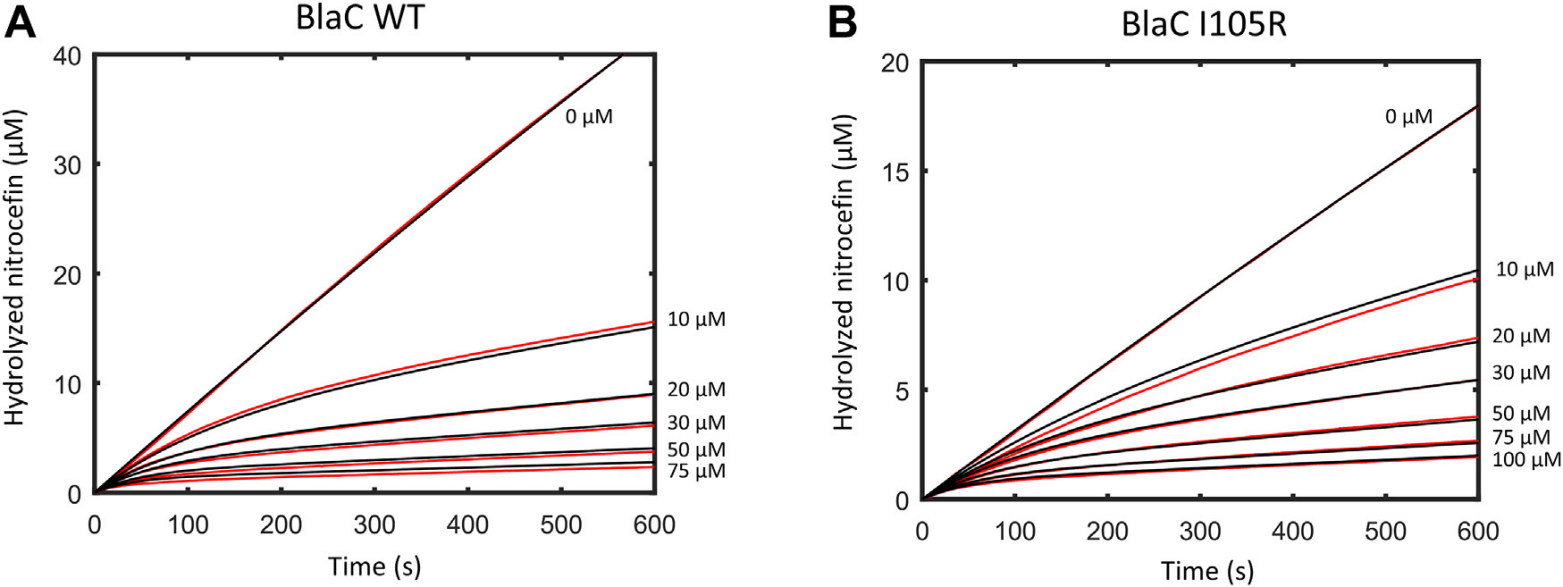
**Inhibition curves of nitrocefin hydrolysis in the presence of increased clavulanic acid concentrations and 2 nM BlaC.***Red lines* represent experimental data, and *black lines* represent simulated data. Measurements were done in 100 mM NaPi buffer, pH 6.4, at 25 °C. *A*, – BlaC wild-type; (*B*) BlaC I105R. WT, wild-type.

Additionally, the avibactam inhibition parameters for all enzymes were derived from the reversible model shown in Equation 3 (24, 25):

(3) $$E+I\;\underset{k_{-1}}{\overset{k_{1}}{⇄}}\;\text{EI}\;\underset{k_{-2}}{\overset{k_{2}}{⇄}}\;EI^{*}$$

where instead of hydrolysis, the slow dissociation reaction of covalently bound avibactam adduct occurs with a rate constant *k*_-2_. Rather than fitting inhibition parameters to the experimental data, which often leads to overfitting (23), the data were simulated by solving the differential equations underlying the kinetic model numerically using GNU Octave software. It is noted that simulations yield a set of rate constants that can describe the observations, but such a set is not necessarily the only solution that can describe the data. We obtained good simulations for both wild-type and mutant enzymes (Figs. 5 and S2), with the values listed in Table 4 (all parameters are listed in Table S1). The data for wild-type and BlaC I105R could be simulated with values for *k*_2_ and *k*_3_ that are very similar between wild type and mutant. However, we needed a *K*_i_ for I105R that is approximately five times higher than for wild-type, implying that the reduced sensitivity of this variant likely originates from diminished affinity for clavulanic acid, rather than enhanced hydrolysis. A similar five-fold increase in *K*_i_ was obtained for clavulanic acid inhibition for TEM-1 Y105G relative to wild-type. Good simulations of data of TEM-1 Y105G inhibition by avibactam could be obtained only with both *K*_i_ and *k*_-2_ values being higher than those of wild type (Fig. S2 and Table 4).

**Table 4:** Rate constants of clavulanic acid hydrolysis and reversible inhibition by avibactam derived from the simulations that were performed using models described in Equations (2), (3) and (2), (3)

	Clavulanic acid			Avibactam		
	*K*_i_^a^ (μM)	*k*_2_ (10^−2^ s^−1^)	*k*_3_ (10^−3^ s^−1^)	*K*_i_^a^ (μM)	*k*_2_ (10^−2^ s^−1^)	*k*_-2_ (10^−3^ s^−1^)
BlaC WT	21	4.5	1.8	2600	6.5	0.1
BlaC I105R	110	4.6	2	14,000	9.5	0.1
CTX-M-14 WT	n.d.	n.d.	n.d.	0.47	6.6	0.3
CTX-M-14 Y105G	n.d.	n.d.	n.d.	50	4.6	0.25
TEM-1 WT	0.85	8	1.8	0.076	5.7	0.2
TEM-1 Y105G	5.15	9.6	3.1	0.42	5	0.75

n.d., not determined.

a *K*_i_ = *k*_-1_/*k*_1_.

### Tyr to Gly substitution promotes the stability of TEM-1

**Figure 6:**
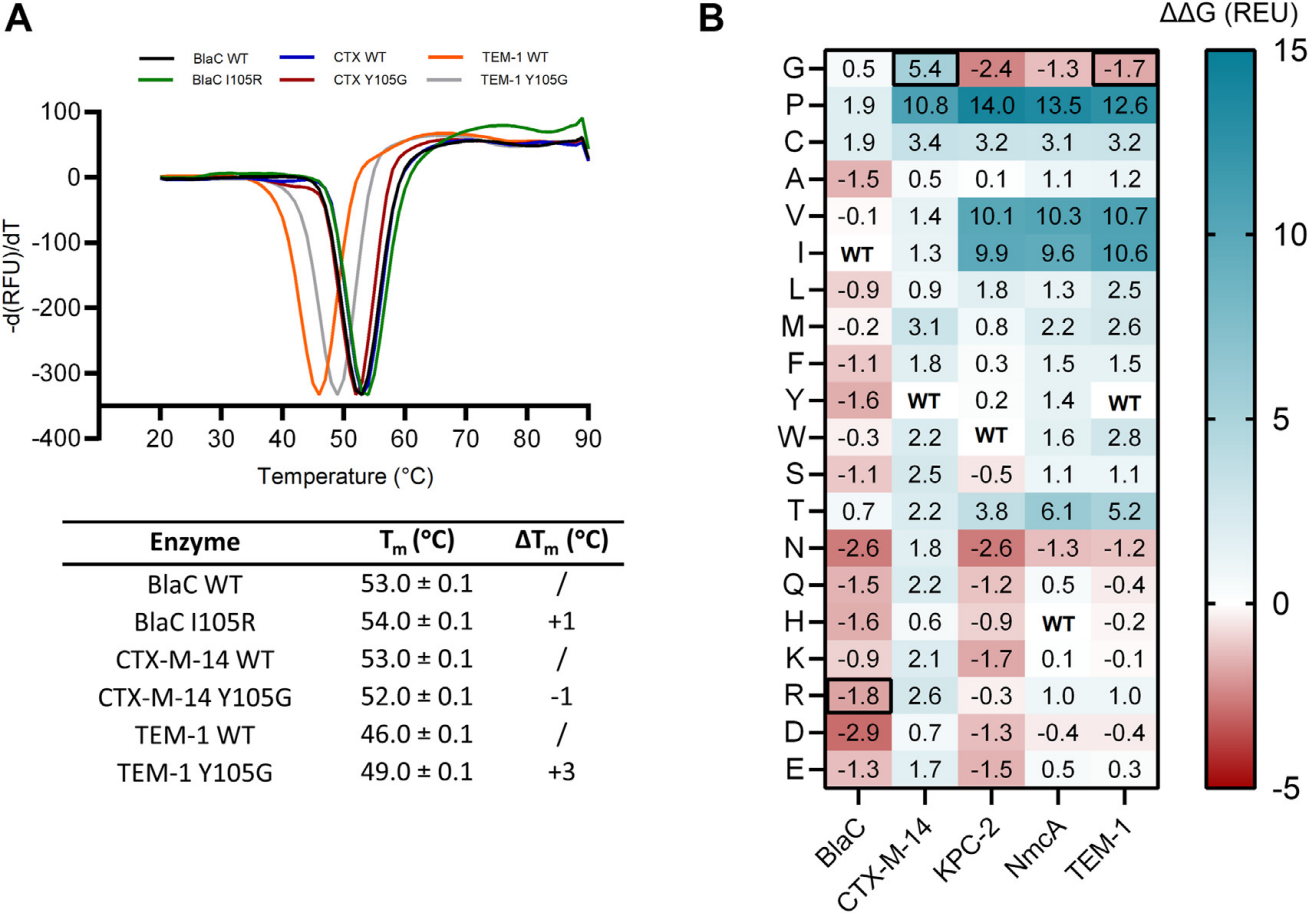
**Stability assessment of several class A β-lactamases.***A*, melting curves of BlaC, CTX-M-14, and TEM-1 enzyme variants. *Top panel* – Negative first derivative of the normalized fluorescence signal; *Bottom panel* – Melting temperatures determined for each variant. Errors represent one standard deviation of the triplicate measurements. *B*, prediction of residue 105 mutational effects on protein stability using the Rosetta Server (26). Calculated free energy differences (ΔΔG) for each mutation in each β-lactamase are given in Rosetta Energy Units (REU) and plotted as a heatmap with *red* representing stabilizing and *blue* destabilizing mutations. The REU values are shown in each cell. BlaC I105R, CTX-M-14 Y105, and TEM-1 Y105G variants are marked with the *black box*. REU is parameterized to approximate kcal/mol (59).

The discrepancy between the decrease in catalytic efficiency (6.6-fold) and MIC value (2-fold) for carbenicillin of TEM-1 Y105G led us to speculate that this mutation may positively impact stability, which could raise the concentration of active enzyme in the bacterial periplasm. Therefore, the melting temperatures (*T*_m_) of all previously purified enzymes were determined from thermal unfolding in the presence of SYPRO orange dye (Fig. 6*A*). Indeed, among all variants, TEM-1 Y105G displayed the highest increase compared to its wild-type counterpart (+3 °C in *T*_m_). The same mutation in CXT-M-14 resulted in a slightly destabilized enzyme (−1 °C in *T*_m_), whereas BlaC I105R showed a modest increase in *T*_m_ (+1 °C). The effect of substitutions on the Gibbs free energy of folding was also calculated using the Rosetta Online Server (26). For this analysis, we also included KPC-2 and NmcA enzymes. The resulting predictions are plotted as a heatmap, with negative values representing favorable mutations (Fig. 6*B*). Interestingly, most of the substitutions in BlaC lead to increased stability, including I105R. Unlike BlaC, the other enzymes mostly display a trend of reduced stability, with hydrophobic residues, such as Val or Ile, or Pro, being highly unfavorable. The mutation Y105G is predicted to stabilize TEM-1, which is in line with the thermal unfolding result (Fig. 6*A*).

### Ile to Arg mutation leads to the active site widening in BlaC

To seek the possible structural changes behind the resistance phenotype of BlaC I105R, we crystallized and solved structures of the resting state enzyme (1.8 Å, PDB 9QI5) and in complex with clavulanic acid (1.8 Å, PDB 9QI6) and avibactam (2.2 Å, PDB 9QI7). Both ligand-bound structures were obtained after 1 h of soaking in crystallization solution, complemented with cryoprotectant and 10 mM inhibitors. Short soaking (∼10 min) did not reveal any detectable density for either of the inhibitors. Crystallization conditions, data collection, and refinement statistics are listed in Table S2.

**Figure 7:**
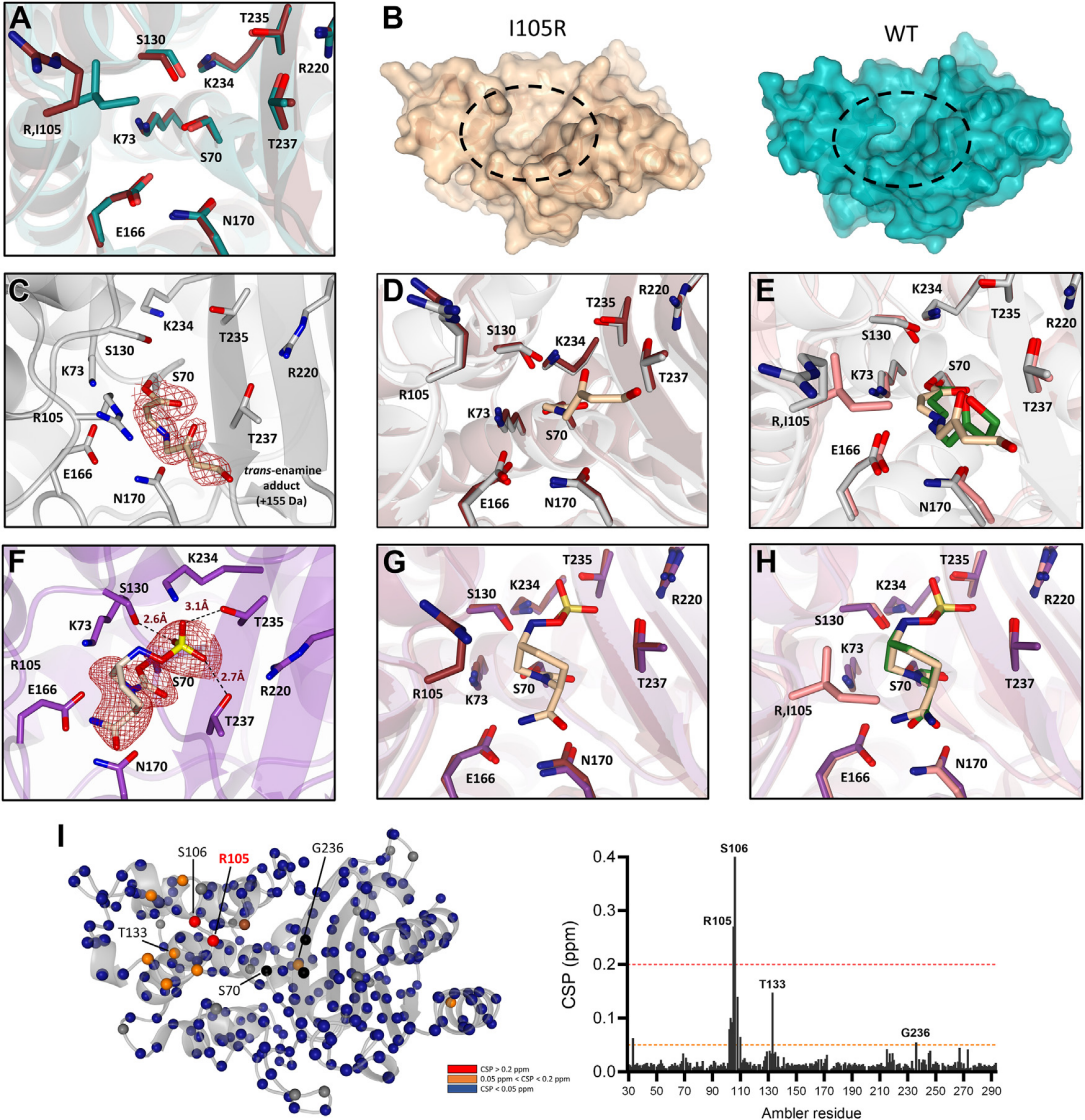
**Structural characterization of BlaC I105R.***A*, superposition of the I105R (PDB 9QI5) and wild-type enzyme (PDB 2GDN (54)) active sites. Side chains of the active site residues are shown as sticks. Upon the mutation of Ile to Arg, the side chain of the gatekeeper residue is pointing outwards. I105R – *ruby*; WT – *teal*. *B*, surface representation of the I105R and WT crystal structures, illustrating broadened access to the active site bestowed by the Ile to Arg mutation. *C*, crystal structure of the I105R enzyme in complex with the *trans*-enamine adduct of clavulanic acid (PDB 9QI6). The 2mF_o_-DF_c_ electron density map (0.9σ) is centered around the clavulanate adduct, which is shown as sticks. *D*, superposition of the resting state I105R and clavulanate-bound enzymes. I105R resting – *ruby*; I105R with clavulanate adduct – *gray*. *E*, superposition of I105R and WT structures with the *trans*-enamine adducts (PDB 6H2C (28)). I105R – *gray*; I105R adduct - *beige*; WT – *salmon*; WT adduct - *green*. *F*, crystal structure of the I105R enzyme in complex with the carbamyl adduct of avibactam (PDB 9QI7). The 2mF_o_-DF_c_ electron density map (1.2σ) is centered around the avibactam adduct, which is shown as sticks. The side chain of Arg105 is not shown due to the missing electron density. *G*, superposition of the resting state I105R and avibactam-bound enzymes. I105R resting – *ruby*; I105R with avibactam adduct – *purple*. *H*, superposition of I105R and wild-type structures in complex with avibactam (PDB 6H2H (28)). I105R – *violet*; I105R adduct – *beige*; WT – *salmon*; WT adduct - *green*. The possible H-bonds are indicated in *dashes* and given in Å. *I*, chemical shift perturbations of backbone amide resonances upon I105R mutation. CSPs are plotted with the color code on the crystal structure (*left*) and as a bar graph per Ambler residue (*right*). *Red*, CSP of ≥0.2 ppm; *orange*, 0.2 ppm > CSP ≥0.05 ppm; *blue*, CSP of <0.05 ppm; *black*, residues could not be assigned or broadened beyond detection; *brown*, residues for which resonances are present in the spectrum of the mutant, but not in the WT spectrum; *gray*, proline residues. *Dotted lines* on the bar graph represent cut-offs for different CSP groups.

Comparison between BlaC I105R and wild-type structures indicates a minor repositioning of the active site residues with one important difference (Fig. 7*A*). The newly introduced arginine at position 105 adopts a conformation that differs from the isoleucine in the WT, moved away from the active site. Consequently, the access to the active site is broadened (Fig. 7*B*). In principle, this would allow for easier accommodation of both substrate and inhibitor molecules, but it can also lead to an augmented dissociation rate of the initial Michaelis complex in case interactions between active residues and substrate or inhibitor have weakened. The crystal structure of BlaC I105R in complex with clavulanic acid revealed the *trans*-enamine adduct of the inhibitor bound to Ser70 (+155 Da, Fig. 7*C*), which is formed after decarboxylation and initial ring opening of the clavulanate (27). In this complex, residues 105, 130, and 166 are slightly shifted in the I105R enzyme compared to the resting state form (Fig. 7*D*). Interestingly, considerably shorter soaking of the wild-type crystal (10 min) was previously found to yield the propionaldehyde ester adduct bound to Ser70 (+70 Da) (28). Alignment with the wild-type structure obtained after 3 min of soaking with clavulanic acid shows different conformations of the *trans*-enamine adduct (PDB 6H2C (28)) (Fig. 7*E*). However, due to the limited resolution of our structure, the difference is likely to be insignificant. In the crystal structure of the I105R variant in the complex with avibactam, the sulfate moiety of the carbamyl adduct of avibactam occupies the carboxylate binding pocket and engages in H-bonding with Ser130, Thr235, and Thr237 (Fig. 7*F*). The electron density of the Arg105 side chain was missing, indicating elevated dynamics of this residue upon binding. Superposition with the resting state structure of I105R and the previously determined structure of wild-type enzyme with avibactam (PDB 62H2 (28)) did not reveal any significant differences in the active site configuration (Fig. 7, *G* and *H*, respectively) (26).

Furthermore, we investigated whether any structural changes in I105R would be detectable in solution using nuclear magnetic resonance (NMR) spectroscopy. Hence, two-dimensional (2D) heteronuclear single-quantum coherence (HSQC) and three-dimensional HNCA spectra were recorded. With the help of previously obtained data for wild-type BlaC (23, 29), 248/251 of non-proline backbone amides could be assigned with confidence. Subsequently, the backbone amide chemical shifts were compared to those of the wild-type BlaC by calculating chemical shift perturbations (CSPs). In agreement with the observation from the crystal structure, only the 102 to 106 loop, harboring the mutation site, experienced large chemical shift perturbations (Fig. 7*I*).

## Discussion

Antimicrobial resistance hinders the efficacy of β-lactam antibiotics and requires constant reevaluation of clinical treatment strategies. One of the primary drivers of resistance is the rapid evolution of β-lactamases, continuously outpacing drug development efforts. Hence, laboratory evolution and fitness studies can serve as valuable tools for forecasting novel β-lactamase variants that might emerge in the clinics and for detailed characterization of the resistance mechanisms (21, 30, 31, 32, 33). Here, we focus on activity and inhibitor profiling of a specific residue in several class A β-lactamases, by employing codon randomization and screening in combination with deep sequencing.

The residue at position 105 gates access to the active site and is speculated to be an important determinant of substrate recognition and stabilization (12, 13, 16, 17). So far, two studies have examined its role in inhibitor resistance with a focus on β-lactam-based inhibitors, such as clavulanic acid or sulbactam (13, 34). In the current study, we also included the non-β-lactam inhibitor avibactam. To have a representative set of class A β-lactamases, we chose five enzymes with four different amino acids at position 105: BlaC (Ile), CTX-M-14 and TEM-1 (Tyr), KPC-2 (Trp), and NmcA (His).

**Figure 8:**
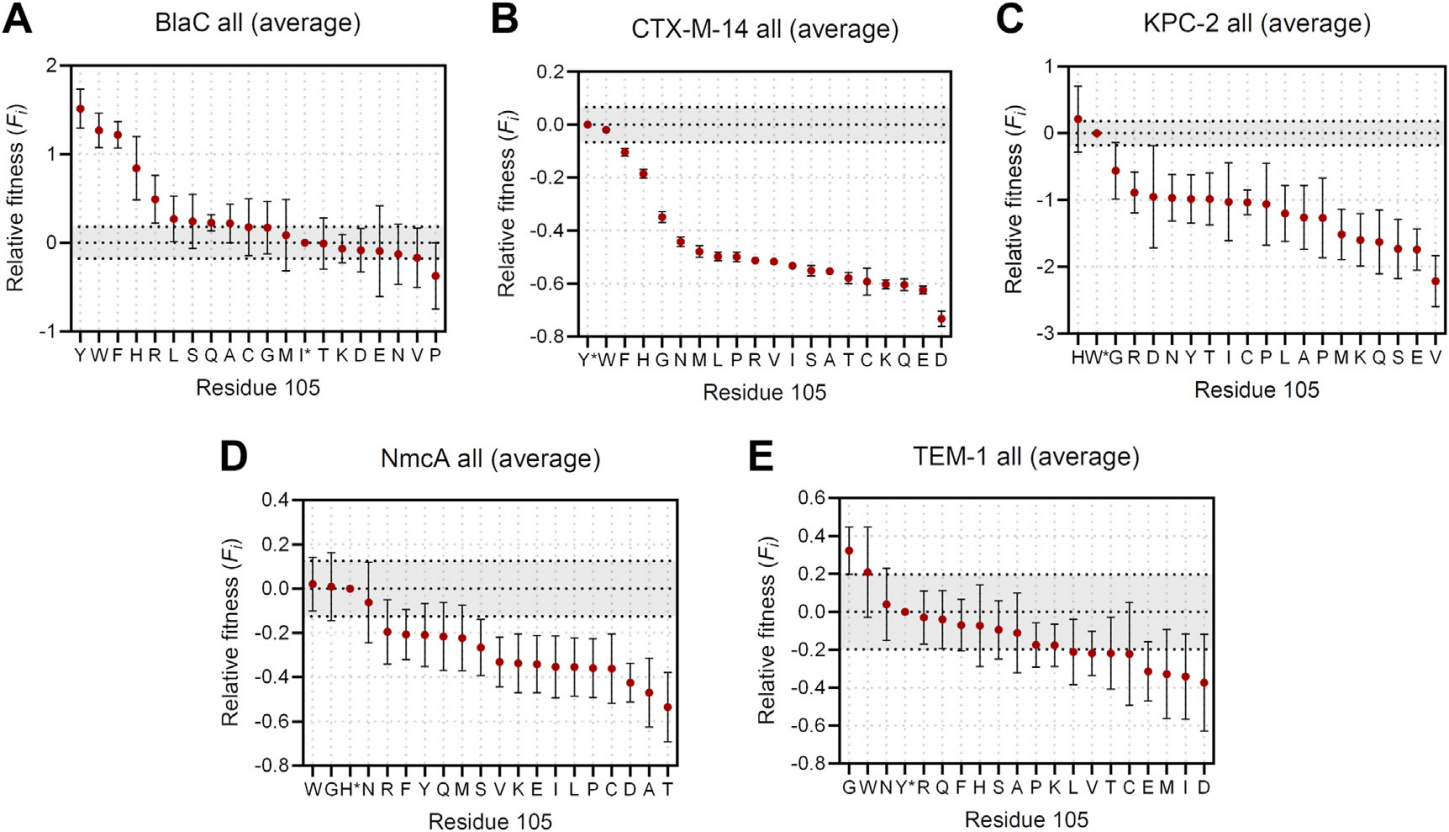
**Relative fitness effects averaged across all tested antibiotics and inhibitors.***A*, BlaC; (*B*) CTX-M-14; (*C*) KPC-2; (*D*) NmcA, and (*E*) TEM-1. *Gray* highlight around zero denotes variability in absolute wild-type fitness (Δ^abs^*F*_*i*_), which was used to determine the significance of relative fitness values. Error bars represent the mean of the average errors or standard deviations of duplicate or triplicate datasets, respectively. The noticeably smaller fitness errors of the CTX-M-14 enzyme are due to the differences in the experimental setup of the screening step.

Aside from BlaC, our fitness data suggest that the replacement of residue 105 has a predominantly negative effect on the fitness of β-lactamases (Fig. 3*A*). This implies that wild-type residues in these enzymes represent the most viable solution and have been evolutionarily optimized. Indeed, when fitness is averaged over all tested substrates and inhibitors, none but one variant has fitness significantly more favorable than the wild-type (TEM-1 Y105G, Fig. 8, *B*–*E*). Moreover, Tyr, Trp, and His are among the best average-performing variants in BlaC, further demonstrating the evolutionary preference of these amino acids at the gatekeeper residue position. It is worth noticing that the high mutation rate associated with plasmid-mediated enzymes likely accelerated the evolution of these residues in the CTX, KPC, and TEM families of enzymes. Concerning β-lactamase inhibitors, we identified at least one significantly beneficial mutation for each β-lactamase, with I105R found for BlaC and substitution to Gly observed in the other β-lactamases (Figs. 3 and S1). Despite high sequence variability among examined class A enzymes, the latter observation suggests that inhibitor-resistant phenotypes with a Gly at this position share a common molecular and structural basis. We first discuss the I105R mutation and then the substitution to Gly105.

The mutation of Ile to Arg in BlaC also conferred noticeably higher bacterial resistance to clavulanic acid and avibactam (Table 2), which motivated us to investigate the biochemical and structural underpinnings of this variant. Kinetic characterization corroborated our previous suggestion about the specificity of this enzyme variant for carbenicillin (Table 3) (18). The positively charged guanidinium group of the arginine side chain might act as a “fishing pole” in attracting negatively charged carbenicillin molecules through the carboxylate group on the C6 atom. Additionally, this favorable electrostatic interaction might be used to lock carbenicillin in an optimal position upon binding. Besides improved activity for carbenicillin, BlaC I105R displayed reduced *in vitro* sensitivity to both clavulanic acid and avibactam (Fig. 4, *A* and *B*). Based on our simulations, the binding affinity (1/*K*_*i*_) for clavulanic acid is approximately 5 times lower (Table 4). Although the structural investigation did not indicate any significant repositioning of the active site residues besides 105 (Fig. 4*A*), we speculate that the larger active site in BlaC I105R causes faster dissociation of the Michaelis complex, thus slowing the initial ring opening of the clavulanate and formation of the acylated species (27). This is in line with the crystallographic observation that the short soaking of I105R protein crystals with clavulanate (∼10 min) did not yield detectable density for the adduct. In contrast, the *trans*-enamine adducts bound to the wild-type BlaC were already present after 3 min of soaking with the inhibitor (PDB 6H2C (28)).

The avibactam-bound structure showed an inward-oriented sulfate moiety of the carbamyl adduct, which is likely engaged in H-bonding with Ser130, Thr235, and Thr237 (Fig. 7*F*). The same orientation was previously observed in the two wild-type structures (PDB 4DF6 (22) and 62H2 (28)) and the structure of CTX-M-15 enzyme with avibactam (PDB 4HBU (35)). In all instances, this is speculated to be a stable inactive conformation of the inhibitor, which is substantially more resistant to hydrolysis than clavulanic acid (22).

As concluded from our fitness data and MIC testing, the substitution of Tyr to Gly in CTX-M-14 and TEM-1 conferred higher bacterial resistance to avibactam (Fig. 3 and Table 2). To our knowledge, these are the first reported avibactam-resistant variants of CTX-M and TEM enzyme families. The *in vitro* characterization suggests that the resistance of these variants predominantly emanates from the weakened binding affinity for avibactam, which is decreased up to 100-fold in the case of CTX-M-14 Y105G (Table 4). Among other class A β-lactamases, the most frequently reported is the resistance to the ceftazidime-avibactam combination found in KPC enzyme variants, described in both clinical (36, 37, 38, 39, 40) and laboratory settings (41, 42, 43, 44). These variants usually carry mutations in the Ω-loop region (residues 162–180) (41, 43, 45). In one such KPC-2 variant, mutation D179N increases Ω-loop flexibility and expands access to the active site, which selectively improves the binding of ceftazidime over the avibactam molecule (43).

Position 105 in TEM-1 β-lactamase has been previously characterized biochemically and structurally, using NMR and molecular dynamics (MD) approaches (12, 46, 47). Our results for Y105G are in agreement with the former data, showing a similar decrease in activity for both penicillin-like substrates (5 to 6-fold) and only a 2-fold drop in MIC value, compared to the wild-type enzyme. The apparent discrepancy between activity and MIC can perhaps be explained by elevated enzyme amounts in the bacterial periplasm, as we found that the Y105G mutation improves stability, at least *in vitro* (Fig. 6). Interestingly, our NMR data for BlaC I105R closely align with those for TEM-1 Y105G, in which moderate to high CSPs are mostly limited to the 102 to 106 loop and residues in and around the SDN motif (129–133) (46). Although crystal structures of the TEM-1 enzyme with substitutions at position 105 have not been reported, the high-resolution structures of the wild-type enzyme in complex with substrate or inhibitor offer a basis to propose a rationale for the structural role of this residue. One of the most noteworthy observations from these acyl-enzyme structures is that the hydroxyphenyl group of Tyr105 moves from its position in the resting state enzyme and interacts with the thiazolidine ring of the penicillin substrate or with the benzene ring of the boronated inhibitor (15, 48). Together with functional characterization of all substitutions at position 105 (12), this led to a proposition that residues that adopt a stable planar conformation between the ligand and the conserved Pro107 create a physical barrier, which facilitates productive substrate positioning. The complete absence of the side chain would, thus, allow for more conformational freedom and lead to non-productive binding of the substrate or inhibitor, which is likely the case for the Y105G variant in TEM-1 and CTX-M-14. Since both enzymes displayed reduced sensitivity for avibactam, we speculate that this displacement effect might be more prominent for bulkier rather than somewhat smaller inhibitors, such as clavulanic acid.

In summation, we have shown that mutations of the residue at position 105 in class A β-lactamases can cause inhibitor-resistant phenotypes. Fitness-based substrate profiling confirmed the evolutionary preference of aromatic or planar residues at this position. However, even long and charged residues, like Arg, can serve as a viable replacement, as demonstrated in BlaC. Although most inhibitor-resistant substitutions reduce enzyme activity, they can have a positive effect on stability, which could lead to elevated enzyme concentrations and enhanced bacterial resistance. In the case of sustained selection pressure of β-lactam antibiotic/inhibitor combinations, we would not be surprised if variants such as TEM-1 Y105G arise in the clinical isolates. The observed reduced sensitivity to both β-lactam and non-β-lactam inhibitors suggests that even future generations of these drugs may exhibit limited efficacy for such variants. Hence, the development of inhibitors that do not rely on the favorable positioning of residue 105 could provide a competitive edge in the race against highly resistant variants of β-lactamases.

## Experimental procedures

### Minimum inhibitory concentration determinations

The MIC was determined for *E. coli* KA797 cells carrying pUK21 plasmids with the *lac* promoter and *tat*- or *sec*-signal sequence upstream of the β-lactamase gene (Fig. S3*A*). Overnight cultures of *E. coli* were inoculated into fresh LB medium with 50 μg/ml kanamycin and incubated at 37 °C until OD_600_ reached 2. Subsequently, cells were pipetted into a honeycomb 100-well plate with the selection antibiotic or antibiotic/inhibitor to an OD_600_ = 0.01. The selection concentration was increased in 2-fold increments. The plate was incubated for 16 h at 37 °C in a Bioscreen C Pro plate reader with continuous shaking, and the OD_600_ was recorded every 15 min. The MIC was defined as the concentration of the selection antibiotic or antibiotic/inhibitor at which no increase in OD_600_ was observed.

### Construction of single-site saturation libraries

pUK21 plasmid libraries of BlaC, CTX-M-14, KPC-2, NmcA, and TEM-1 β-lactamases with single codon randomization at position 105 were initially constructed using mutagenic primers carrying NNS or NNK degeneracy (Table S3) and the QuikChange method (Agilent). However, due to the high background of the wild-type codon, libraries of BlaC, CTX-M-14, NmcA, and TEM-1 enzymes were prepared again with the same mutagenic primers and two overlapping PCR reactions. PCR products were gel-purified using Illustra GFX PCR DNA and Gel Band Purification Kit (Cytiva) and subsequently ligated using Gibson Assembly (49). The reaction was done at 50 °C for 1 h, and the product was purified with the Monarch PCR & DNA cleanup kit (New England Biolabs). The resulting plasmid libraries were introduced in *E. coli* KA797 cells by electroporation, which were plated on nonselective lysogeny broth (LB) agar plates containing 50 μg/ml kanamycin (vector selection marker) and incubated for 16 h at 37 °C. Each transformation yielded more than 10^5^ transformants. Colonies from each plate were scraped with 10 ml of LB broth, vortexed, and 1 ml of each suspension was used for plasmid isolation using GeneJET Plasmid Miniprep Kit (Thermo Fischer Scientific).

### Screening of library mutants against **β**-lactam antibiotics and inhibitors

All selection experiments were performed in *E. coli* strain KA797 (50). *E. coli* cells were transformed by electroporation with 100 ng of each plasmid library, plated on nonselective media (LB-agar with 50 μg/ml kanamycin), and incubated overnight at 37 °C. Each transformation yielded more than 10^7^ transformants. Cells were recovered from agar plates with 5 ml of LB broth and diluted 1:10, after which OD_600_ was measured in triplicate. These cultures were used to inoculate 200 μl of nonselective media (LB broth with 50 μg/ml kanamycin) and 200 μl of selective media (LB broth with 50 μg/ml of kanamycin and β-lactam antibiotic with or without inhibitor) in honeycomb 100-well plates to have an OD_600_ = 0.01. For each β-lactam antibiotic or antibiotic/inhibitor pair, the selection was done at previously determined MIC values for the wild-type enzyme. The plate was incubated for 5 h at 37 °C in a Bioscreen C Pro plate reader with continuous shaking, and the OD_600_ was recorded every 15 min. Each selection experiment was performed in triplicate. Afterward, the plate was put on ice for 5 min to stop the growth. The incubation time was chosen to obtain significant selection while maintaining a sufficient population size relative to the library diversity and avoiding the stationary phase. The cultures were centrifuged, washed twice by resuspension in 1 ml of LB broth, then centrifuged again and resuspended in 2 ml LB broth with 50 μg/ml kanamycin, and incubated overnight to saturation at 37 °C. The next morning, plasmid libraries were harvested using the GeneJET Plasmid Miniprep Kit (Thermo Fischer Scientific). Due to the high variability in sequencing results and subsequently large fitness errors, the selection of samples BlaC (ceftriaxone and clavulanic acid), CTX-M-14 (all antibiotics and inhibitors), NmcA (ampicillin and carbenicillin), and TEM-1 (ampicillin and carbenicillin) was performed as described previously (18).

### Preparation of amplicons and deep sequencing

Samples for Illumina sequencing were prepared by PCR using both selected and unselected libraries as templates. The purity of amplicons was assessed with agarose gel electrophoresis. Amplicons were barcoded using universal tail sequences and sequenced by Leiden Genome Technology Center on an Illumina MiSeq sequencer. Samples were sequenced paired-end 300-bp using 600 cycle v3 sequencing reagents. FASTQ files were generated using bcl2fastq v2.20.

### Analysis of deep sequencing data

The sequencing data were analyzed using the Galaxy open web-based platform (51, 52, 53) and custom Python 3.1 scripts. Reads were filtered for quality score (reads with >10% of base calls with a quality score less than 20, or probability of error = 10^−2^, were discarded) and length (only accepting reads with 146 nt for BlaC, 112 nt for CTX-M-14, 116 nt for KPC-2, 111 nt for NmcA, and 101 nt for TEM-1). Identical reads were collapsed into a single read in FASTA format, and the frequency of each variant under each condition was determined.

Relative fitness values were determined to be significantly positive or negative based on Equations 4 and 5, respectively:

(4) Fi–(ΔFi+ΔabsFWT)>0

(5) Fi+(ΔFi+ΔabsFWT)<0

where *F*_*i*_ stands for the relative fitness of a specific variant, Δ*F*_*i*_ for its error between duplicate or triplicate datasets, and ^abs^Δ*F*_*WT*_ for a variation between duplicate or triplicate absolute fitness values of the wild type (difference in the ratio of selected vs. unselected reads, see Equation 1). Sequencing counts following quality filtering and calculated fitness values are available in the Supporting Data.

### Site-directed mutagenesis

Arginine and glycine substitutions of BlaC, CTX-M-14, and TEM-1 enzymes were introduced in their corresponding genes by the QuikChange method (Agilent) and verified by Sanger sequencing. All mutagenic primers are listed in Table S3.

### Protein production and purification

Recombinant proteins were produced in *E. coli* BL21 (DE3) pLysS cells transformed with pET28a plasmids carrying the β-lactamase gene with an N-terminal His tag and TEV cleavage site (Fig. S3*B*). Protein production and purification were performed as described previously (23), but omitting the size exclusion chromatography step. The purity of protein samples was assessed with SDS-PAGE (≥95%). Pure protein samples were aliquoted, flash-frozen in liquid nitrogen, and stored at −80 °C in 100 mM sodium phosphate buffer (pH 6.4) or 100 mM MES buffer (pH 6.4).

### Enzyme kinetics

Steady-state kinetic parameters were determined using nitrocefin (BioVision), ampicillin (Serva), and carbenicillin (Sigma-Aldrich) as substrates. Measurements were performed in triplicate in a 10 mm QS High Precision cell (Hellma Analytics), using a PerkinElmer Lambda 1050+ UV-Vis spectrometer thermostated at 25 °C. Nitrocefin conversion was monitored at 486 nm (Δε = 18,091 M^−1^cm^−1^) for 3 min in the presence of 2 nM BlaC, 0.2 or 0.5 nM CTX-M-14, and 0.25 or 2 nM TEM-1 enzymes. Ampicillin hydrolysis was followed at 235 nm (Δε = 861 M^−1^cm^−1^) for 3 min in the presence of 10 nM BlaC, 2 or 10 nM CTX-M-14, and 0.5 nM TEM-1. Carbenicillin hydrolysis was recorded at 235 nm (Δε = 938 M^−1^cm^−1^) for 3 min with 50 nM BlaC, 2 or 10 nM CTX-M-14, and 2 nM TEM-1. Substrate concentration was varied from 10 to 600 μM (nitrocefin), 5 to 600 μM (ampicillin), and 2.5 to 200 μM (carbenicillin). Kinetic experiments with CTX-M-14 and TEM-1 enzymes were done in the presence of 10 μM BSA. All measurements were done in 100 mM phosphate buffer (pH 6.4). GraphPad Prism 10.1 was used to fit initial reaction velocities to the Michaelis-Menten equation (Equation 6):

(6) $$v_{i}=\frac{k_{\text{cat}\;}\left[E\right]\left[S\right]}{K_{M}^{\text{app}}+\left[S\right]}$$

Where *v*_*i*_ is the initial reaction velocity, [S] is the initial substrate concentration, [E] is the total enzyme concentration, and *k*_cat_ and *K*_M_^app^ are the Michaelis-Menten parameters. The *K*_M_ is indicated as apparent because regular Michaelis-Menten kinetics do not apply to the two-step hydrolysis reaction (21). Michaelis-Menten plots of all analyzed variants for all three substrates are shown in Figure S4.

### *In vitro* inhibition studies

Inhibition assays of BlaC enzymes (2 nM) were performed in the presence of various clavulanic acid (10–100 μM) and avibactam (100 μM–5 mM) concentrations with a constant nitrocefin concentration (125 μM with clavulanic acid and 150 μM with avibactam). Inhibition assays of CTX-M-14 variants (0.2 nM) were carried out with avibactam (0.25–10 μM) and 150 nM nitrocefin. Inhibition assays of TEM-1 enzymes (0.25 or 0.2 nM) were done in the presence of clavulanic acid (0.25–5 μM) and avibactam (50 nM–5 μM) with 200 μM nitrocefin. Inhibition experiments with CTX-M-14 and TEM-1 enzymes were done in the presence of 10 μM BSA. All measurements were performed in phosphate buffer (100 mM NaPi, pH 6.4) at 25 °C. The absorbance at 486 nm (Δε = 18,091 M^−1^ cm^−1^) was followed for 10 min, and the amount of the hydrolyzed product was expressed relative to the control (no inhibitor). Errors represent one standard deviation of the triplicate measurement.

The clavulanic acid and avibactam inhibition parameters for BlaC, CTX-M-14, and TEM-1 enzymes were obtained from simulating data using GNU Octave 6.2.0 and numerical simulations of the differential equations derived from the model described with Equations (7), (8), (9):

(7) $$N+E\;\underset{k_{-a}}{\overset{k_{a}}{⇄}}\;\text{NE}\;\overset{k_{b}}{\to }\;I_{1}\overset{k_{c}}{\to }P_{1}+E$$

(8) $$C+E\;\underset{k_{-1}}{\overset{k_{1}}{⇄}}\;\text{CE}\;\overset{k_{2}}{\to }\;I_{2}\overset{k_{3}}{\to }P_{2}+E$$

(9) $$A+E\;\underset{k_{-1}}{\overset{k_{1}}{⇄}}\;\text{AE}\;\underset{k_{-2}}{\overset{k_{2}}{⇄}}\;I_{3}$$

where (N) stands for nitrocefin, (E) for an enzyme, (C) for clavulanic acid, (A) for avibactam, (NE), (CE) and (AE) for noncovalent complexes, (I_i_) for covalent species, and (P_i_) for products. An example script for the simulation of the clavulanic acid inhibition curves is provided in the Supporting file. Equation 8 is equivalent to the conversion model shown in Equation 2, and Equation 9 is equal to the reversible model shown in Equation 3.

### Thermal shift assay

The thermal stability of the proteins was assessed with a thermal shift assay using a CFX96 Touch Real-time PCR Detection System (Bio-Rad). The fluorescence signal of SYPRO Orange dye was followed in the presence of 10 μM enzymes in 100 mM phosphate buffer (pH 6.4). The temperature was increased from 20 to 90 °C with a 1 °C increment, and the samples were incubated for 1 min at each temperature before detection of the fluorescence signal. Melting temperatures (*T*_m_) were determined from the derivatives of the obtained curves. Errors in the reported values represent one standard deviation of the mean of triplicate measurements.

### Computational prediction of stabilizing mutations

The effect of residue 105 substitutions on protein stability was assessed using Rosetta Online Server and the point mutation (PM) protocol (26). The wild-type structures of all enzymes (BlaC, PDB 2GDN (54); CTX-M-14, 1YLT (55); KPC-2, 2OV5 (56); NmcA, 1BUE (57), and TEM-1, 1BTL (58)) were used as a starting point and relaxed to a low-energy state before mutagenesis. At the selected amino acid position, the Rosetta energy function approximates the Gibbs free energy of folding upon mutation to all other 19 residues. The ΔΔG values are reported in Rosetta Energy Units (REU), which are parameterized to be on a similar scale as kcal/mol (59).

### Crystallization

Crystallization conditions for BlaC I105R at a concentration of 10 mg/ml were screened for by the sitting-drop method using the BCS and Morpheus (Molecular Dimensions) screens at 20  °C with 200 nl drops with a 1:1 protein to screening condition ratio. Based on initial hits in the form of needles, the crystal growth was further optimized by varying the concentrations of sodium acetate, zinc chloride, and pH, yielding suitable crystals within a month. The exact formulation of the conditions can be found in Table S2. The structures of BlaC I105R with ligands were obtained by soaking the crystals in the corresponding mother liquor supplemented with 10 mM clavulanic acid or avibactam for 1 h. All crystals were then mounted on cryo-loops in mother liquor with an additional 25% glycerol for cryoprotection.

### X-ray data collection and structure solving

Diffraction data were collected at the European Synchrotron Radiation Facility (Grenoble, France). Diffraction data were recorded with a wavelength of 0.87Å on a Dectris Eiger2_9M detector for I105R and I105R_AVI, and 0.97Å with a Dectris Eiger1_4M detector for I105R_CA. The resolution cutoff was determined based on completeness and CC1/2 values. The data were scaled using Aimless (60). The structures were solved by molecular replacement using MOLREP from the CCP4 suite (61) using PDB entry 2GDN (54) as a search model. All structures, apart from I105R with avibactam, contained two protein molecules in an asymmetric unit. Subsequently, building and refinement were performed using Coot and REFMAC (61). The final refinement for the I105R_CA structure was performed with the PDB REDO web server (62, 63). Structure validation showed a RamaZ (63) score of −0.11, −0.22, and 1.6 for I105R, I105R_CA, and I105R_AVI, respectively; 98% of all residues are within the Ramachandran plot-favored regions, with residues Cys69 and Arg220 being the outliers in all the structures. Data collection and refinement statistics can be found in Table S2.

### Nuclear magnetic resonance spectroscopy experiments

^1^H-^15^N TROSY-HSQC and 3D HNCA spectra for BlaC I105R were recorded at 25 °C using a Bruker AVIII HD 850 MHz spectrometer equipped with a TCI cryoprobe. The sample contained ∼0.6 mM [^15^N] or ∼0.5 mM [^13^C,^15^N] BlaC I105R in 100 mM sodium phosphate (pH 6.4) and 8% D2O. Data were processed with Topspin 4.0.7 (Bruker Biospin) and analyzed using CCPNmr Analysis V2.4.2 (64). Spectra were compared to the HSQC and HNCA spectra of the wild-type BlaC (23, 29), and average chemical shift perturbations (CSPs, Δ*δ*) of backbone amides were calculated using Equation 10:

(10) $$\Delta \delta =\sqrt{\frac{1}{2}\;\left[{\Delta \delta_{1}+\left(\frac{{\Delta \delta }_{2}}{5}\right)}^{2}\right]}$$

where Δ*δ*_1_ and Δ*δ*_2_ are the differences in chemical shifts in ppm in spectra of the mutant and wild-type enzymes for ^1^H and ^15^N, respectively. An overlay of ^1^H-^15^N TROSY-HSQC spectra for BlaC I105R and wild-type is shown in Figure S5.

## Data availability

Raw sequencing files are available through NCBI SRA, under BioProject accession PRJNA1267082. Sequencing counts following quality filtering and calculated fitness values are available in the Supporting Data. Custom Python 3.1 scripts, which were used for variant count, are available upon request. NMR chemical shift assignment of BlaC I105R has been submitted to the Biological Magnetic Resonance Data Bank (BMRB) and can be accessed under BMRB ID 53203. The crystal structures and data files of free form BlaC I105R and bound to clavulanic acid and avibactam have been submitted to the Protein Data Bank (PDB) and can be accessed through codes 9QI5, 9QI6, and 9QI7, respectively.

## Supporting information

This article contains supporting information.

## Conflict of interest

The authors declare that they have no conflicts of interest with the contents of this article.
