# Supplementary material for: A glycine at position 105 leads to clavulanic acid and avibactam resistance in class A β-lactamases
Source: J Biol Chem. 2025 Jun 6;301(7):110347. doi: 10.1016/j.jbc.2025.110347 (PMC12274840; doi:10.1016/j.jbc.2025.110347)
Supplement: Supplementary Data 1 [file mmc1.pdf]

Supporting Information for

A glycine at position 105 leads to clavulanic acid and avibactam resistance in class A β-lactamases

Marko Radojković[1], Aleksandra Chikunova[1,2], Saar F. Koene[1], Monika Timmer[1], Sivanandam V. Natarajan[1], Aimee L. Boyle[1,3] and Marcellus Ubbink[1,*]

[1]Leiden Institute of Chemistry, Leiden University, Einsteinweg 55, 2333 CC Leiden, The Netherlands
[2]The Netherlands Cancer Institute, Plesmanlaan 121, 1066 CX Amsterdam, The Netherlands
[3]School of Chemistry, University of Bristol, BS8 1TS, Bristol, United Kingdom

*Corresponding author, e-mail: m.ubbink@chem.leidenuniv.nl

Supporting Tables

Supporting Figures

Supporting References

SUPPORTING TABLES

Table S1. The parameters of BlaC, CTX-M-14, and TEM-1 inhibition by clavulanic acid and avibactam used for the simulations shown in Figures 5 and S2. The models are described in Equations 7, 8, and 9. Note that some parameters are correlated and should be considered as indicative. The enzyme and nitrocefin concentrations were 2 nM and 125 or 150 µM (BlaC), 0.2 nM and 200 µM (CTX-M-14), and 0.2 or 0.25 nM and 200 µM (TEM-1), respectively. The parameters are derived by fitting simulations to experimentally obtained inhibition curves in 100 mM NaPi buffer, pH 6.4, at 25 °C. The script for the simulation of inhibition curves in the presence of increased clavulanic acid concentrations is provided at the end of this file. WT – wild-type.

	Clavulanic acid							
	K_D (µM)[a]	k_a (µM^{-1}s^{-1})	k_b (s^{-1})	k_c (s^{-1})	K_i (µM)	k_1 (µM^{-1}s^{-1})	k_2 (10^{-2} s^{-1})	k_3 (10^{-3} s^{-1})
BlaC WT	160	1.05	2000	56	21	1	4.5	1.8
BlaC I105R	150	0.18	2000	57	110	1	4.6	2
TEM-1 WT	60	48	8400	830	0.85	0.9	8	1.8
TEM-1 Y105G	50	10	1000	154	5.15	0.9	9.6	3.1
	Avibactam							
	K_D (µM)[a]	k_a (µM^{-1}s^{-1})	k_b (s^{-1})	k_c (s^{-1})	K_i (µM)	k_1 (µM^{-1}s^{-1})	k_2 (10^{-2} s^{-1})	k_{-2} (10^{-3} s^{-1})
BlaC WT	120	1	1900	82.5	2600	1	6.5	0.1
BlaC I105R	150	0.18	1900	40	14,000	1	9.5	0.1
CTX-M-14 WT	50	60	3800	545	0.47	1	6.6	0.3
CTX-M-14 Y105G	760	50	4200	460	50	1	4.6	0.25
TEM-1 WT	50	45	8000	1040	0.076	1	5.7	0.2
TEM-1 Y105G	50	10	1000	95	0.42	1	5	0.75

[a] $K_D = k_{-a}/k_a$

Table S2. Crystallization conditions, data collection, and refinement statistics for the BlaC I105R structures.

**Data collection**	**I105R**	**I105R_CA**	**I105R_AVI**
**PDB ID**	9QI5	9QI6	9QI7
Conditions	0.024 M ZnCl2<br>22 %v/v PEGSM<br>0.2 M Na Acet, pH 4.08	0.067 M ZnCl2<br>22 %v/v PEGSM<br>0.2 M Na Acet, pH 4.46	0.06 M ZnCl2<br>22 %v/v PEGSM<br>0.2 M Na Acet, pH 4.74
Resolution (Å)	44.62-1.80 (1.84-1.80)	53.93-1.80 (1.84-1.8)	80.42-2.20 (2.27-2.20)
Space group	P212121	P212121	C2221
Unit cell *a*, *b*, *c* (Å)	39.16, 40.97, 267.49	39.49, 41.35, 269.67	110.66, 117.07, 51.06
α, β, γ	90, 90, 90	90, 90, 90	90, 90, 90
CC1/2	99.5 (58.9)	99.4 (72.0)	99.8 (45.3)
R pim (%)	6.8 (45)	6.8 (39)	5.2 (63.9)
<*I*/σ>	8.2 (1.6)	8.6 (1.8)	12.3 (1.5)
Completeness (%)	98.6 (99.9)	99.8 (99.9)	99.9 (99.8)
Multiplicity	1.8 (1.9)	5.5 (5.8)	9.2 (9.6)
Unique reflections	40588 (2018)	42289 (2409)	17265 (1465)
**Refinement**			
Atoms protein/ions/ligands/water	4052/1/30/261	3989/5/73/402	1984/2/38/34
B-factors protein/ions/ligands/water (Å^2)	25/82/35/33	22/57/37/29	31/107/64/48
Rwork/Rfree (%)	17/21	18/22	19/24
Bond lengths RMSZ/RMSD (Å)	0.536/0.0136	0.530/0.0315	0.667/0.0106
Bond angles RMSZ/RMSD (°)	0.836/1.49	0.826/1.77	0.961/1.96
Ramachandran plot preferred/outliers	487/4	486/5	244/2
RamaZ score	-0.11	-0.22	-1.60
Clash score	1.73	4.34	3.74
MolProbity score (percentile)	0.93 (100th)	1.21 (99th)	1.16 (100th)

Table S3. Primer sequences used in this study.

**Cloning primers**	
TEM-1_pUK21_F	TTTAACTTTAAGAAGGAGATATACCATGAGTATTCAACATTTCCGTGTCG
TEM-1_ pUK21_R	GATCGTCAGTGGTGGTGGTGGTGGTGCCAATGCTTAATCAGTGAGGCA
pUK21_C1_F	CACCACCACCACCACCACTGACGATC
pUK21_C1_R	GGTATATCTCCTTCTTAAAGTTAAACAAACC
pelB_pUK21_R	GGAGCAGCAGACCAGCAGCAGCGGTCGGCAGCAGGTATTTCATGGTATATCTCCTTCTTAAAGTTAAACAAACC
pelB_NmcA_F	TGTGCTGGTCTGCTGCTCCTCGCTGCCCAGCCGGCGATGGCCAATACCAAAGGTATTGATGAAATCAAGAACC
NmcA_pUK21_R	GATCGTCAGTGGTGGTGGTGGTGGTGTTTCAGATTATCGATGGCAATACG
non_cod_pUK21_F	GCATCCGCTTACAGACAAGCTGTG
non_cod_pUK21_R	CACAGCTTGTCTGTAAGCGGATGC
**Codon randomization primers**	
BlaC_105_NNS_F	ATACCAGTGATGATATCCGTAGCNNSAGTCCGGTTGCACAGCAGCATGTTC
BlaC_105_NNS_R	GAACATGCTGCTGTGCAACCGGACTSNNGCTACGGATATCATCACTGGTAT
CTX_105_NNS_F	TCAAACCGGCAGATCTGGTTAACNNSAATCCGATTGCAGAAAAACATG
CTX_105_NNS_R	CATGTTTTTCTGCAATCGGATTSNNGTTAACCAGATCTGCCGGTTTGA
KPC-2_105_NNS_F	TATGGTAAAAATGCACTGGTTCCGNNSTCACCGATTAGCGAAAAATATCTG
KPC-2_105_NNS_R	CAGATATTTTTCGCTAATCGGTGASNNCGGAACCAGTGCATTTTTACCATA
NmcA_105_NNS_F	TATAACACCCGCAGCCTGGAATTTNNSAGCCCGATTACCACCAAATACAAAG
NmcA_105_NNS_R	CTTTGTATTTGGTGGTAATCGGGCTSNNAAATTCCAGGCTGCGGGTGTTATA
TEM-1_105_NNK_F	TCTCAGAATGACTTGGTTGAGNNKTCACCAGTCACAGAAAAGCATC
TEM-1_105_NNM_R	GATGCTTTTCTGTGACTGGTGAMNNCTCAACCAAGTCATTCTGAGA
**Amplicon primers**	
NGS_BlaC_105_F	GATGTGTATAAGAGACAGTGATTACCTATACCAGTGATGATATCCG
NGS_BlaC_105_R	CGTGTGCTCTTCCGATCTCAGATCGGCCAGCAGCAGATTG
NGS_CTX_105_F	GATGTGTATAAGAGACAGCAGAGCGAAACCCAGAAACAGC
NGS_CTX_105_R	CGTGTGCTCTTCCGATCTCCAGGGTCATTGTACCATTCACAT
NGS_KPC-2_105_F	GATGTGTATAAGAGACAGCGTTATGGTAAAAATGCACTGG
NGS_KPC-2_105_R	CGTGTGCTCTTCCGATCTGCTGCATTATCGCTATACTGAACG
NGS_NmcA_105_F	GATGTGTATAAGAGACAGGGATAATCGTCTGAATCTGAACCAG
NGS_NmcA_105_R	CGTGTGCTCTTCCGATCTATATCACCCAGGCTCATACCATTATC
NGS_TEM-1_105_F	GATGTGTATAAGAGACAGTCGCCGCATACACTATTCTCAG
NGS_TEM-1_105_R	CGTGTGCTCTTCCGATCTCAGCACTGCATAATTCTCTTACTG
**Site-directed mutagenesis primers**	
BlaC_I105R_F	GTGATGATATCCGTAGCCGTAGTCCGGTTGCACAG
BlaC_I105R_R	CTGTGCAACCGGACTAAAGCTACGGATATCATCAC
BlaC_I105G_F	GTGATGATATCCGTAGCGGTAGTCCGGTTGCACAG
BlaC_I105G_R	CTGTGCAACCGGACTACCGCTACGGATATCATCAC
CTX_Y105G_F	CAAACCGGCAGATCTGGTTAACGGGAATCCGATTGCAGAAAAACATGTG
CTX_Y105G_R	CACATGTTTTTCTGCAATCGGATTCCCGTTAACCAGATCTGCCGGTTTG
CTX_Y105R_F	CAAACCGGCAGATCTGGTTAACCGCAATCCGATTGCAGAAAAACATGTG
CTX_Y105R_R	CACATGTTTTTCTGCAATCGGATTGCGGTTAACCAGATCTGCCGGTTTG
TEM-1_Y105G_F	TCTCAGAATGACTTGGTTGAGGGGTCACCAGTCACAGAAAAGCATC
TEM-1_Y105G_R	GATGCTTTTCTGTGACTGGTGACCCCTCAACCAAGTCATTCTGAGA
TEM-1_Y105R_F	TCTCAGAATGACTTGGTTGAGAGGTCACCAGTCACAGAAAAGCATC
TEM-1_Y105R_R	GATGCTTTTCTGTGACTGGTGACCTCTCAACCAAGTCATTCTGAGA

SUPPORTING FIGURES

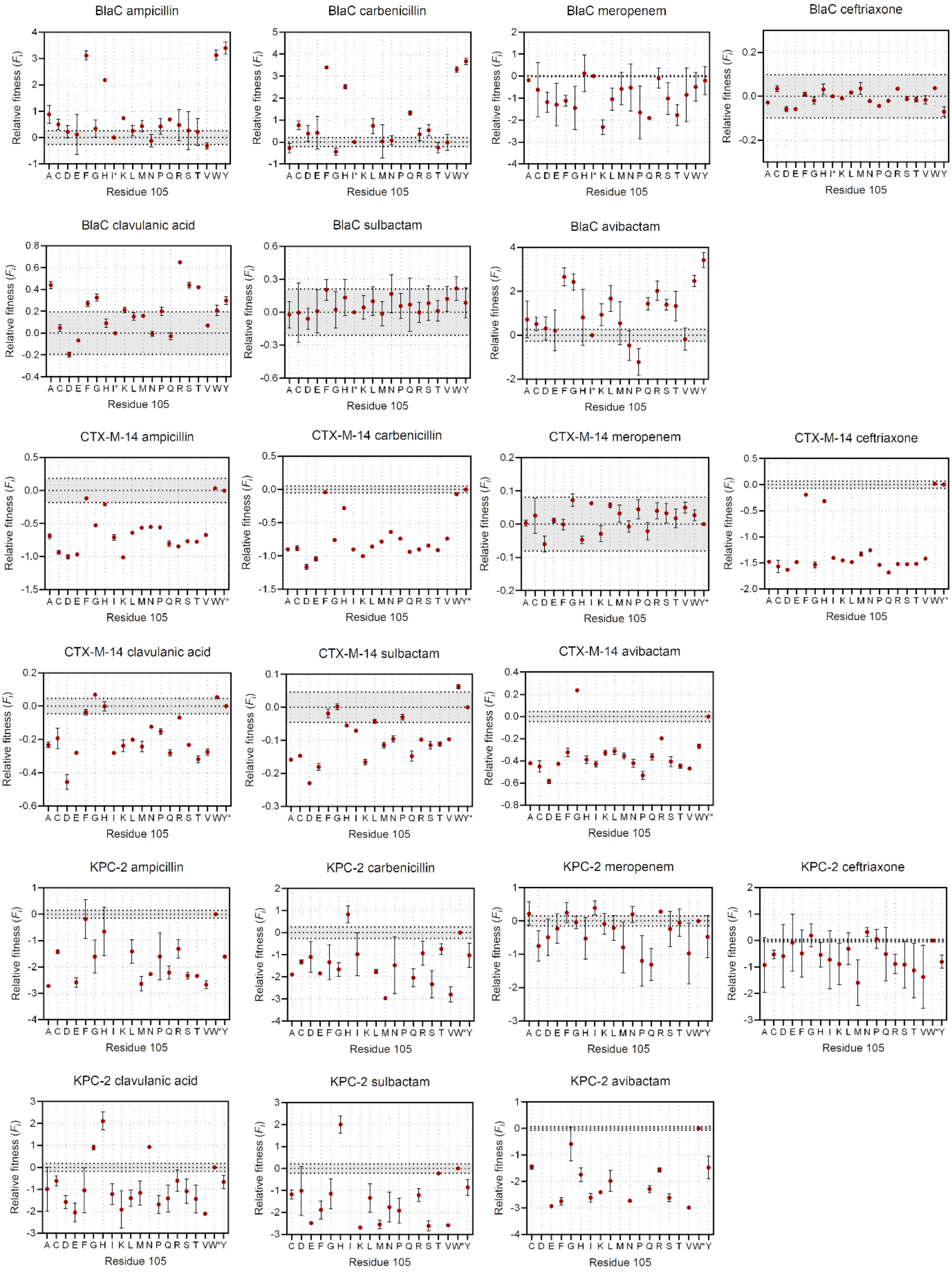

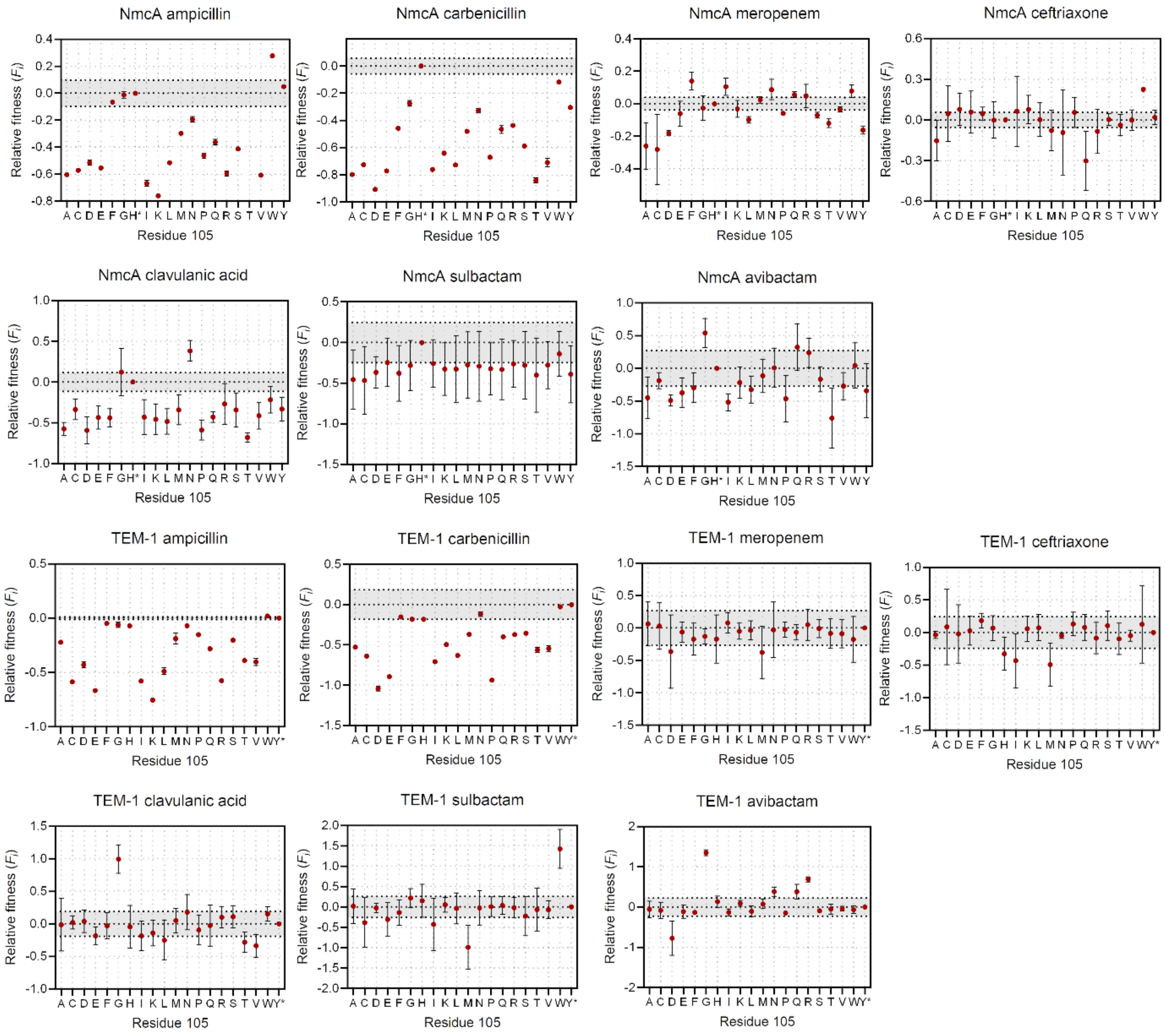

Figure S1. Complete fitness data of all five β-lactamases inspected in this study. Fitness values relative to the wild-type were calculated for all library variants at position 105 according to Equation 1 in the main text, and plotted for various substrates and carbenicillin/inhibitor combinations. Gray highlight around zero denotes variability in absolute wild-type fitness ($\Delta^{abs}F_i$), which was used to determine the significance of relative fitness values (see Materials & Methods in the main text). Error bars represent the average error or standard deviation of duplicate or triplicate datasets, respectively.

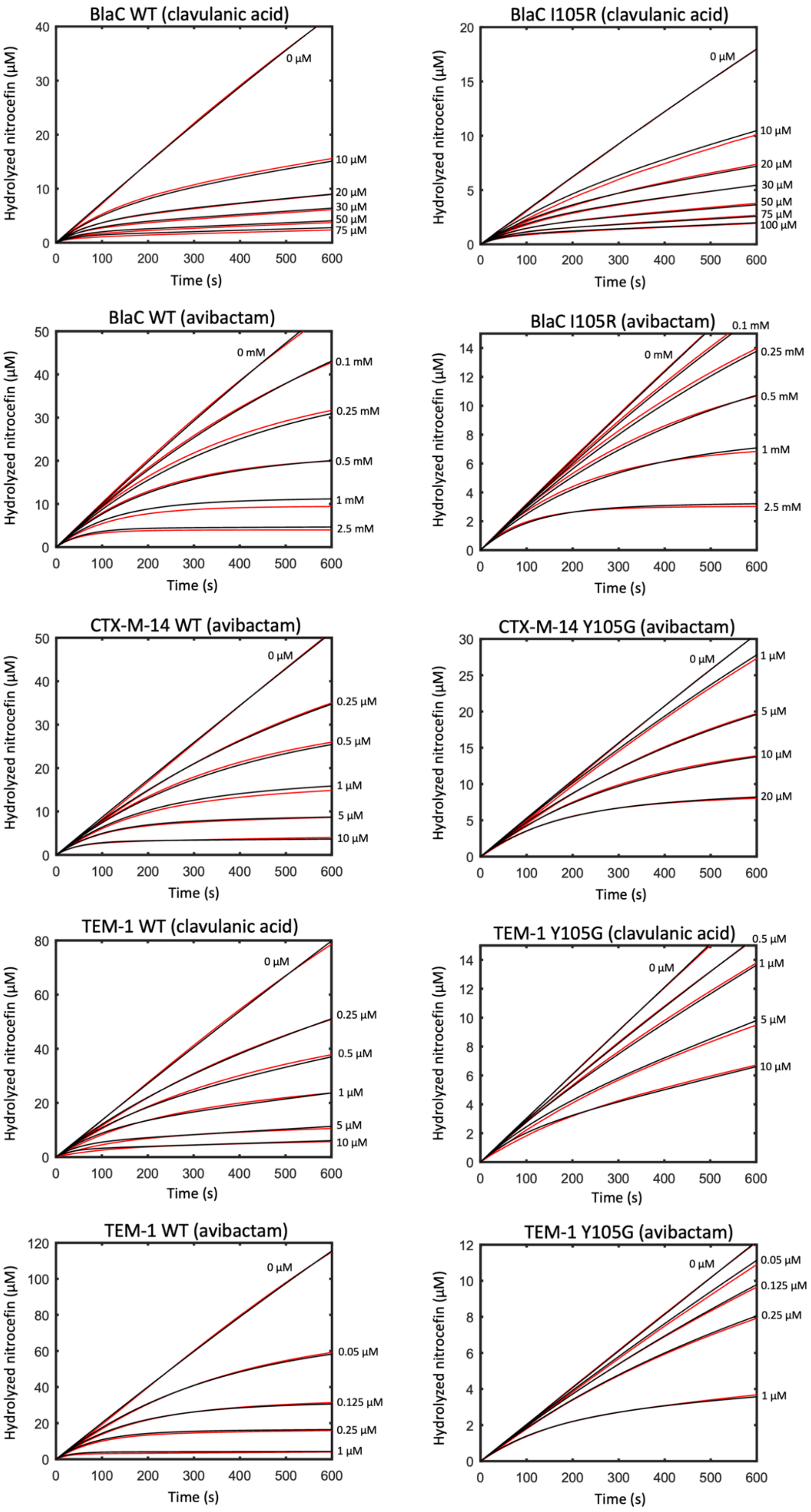

Figure S2. Inhibition curves of nitrocefin hydrolysis in the presence of increased clavulanic acid or avibactam concentrations and 2 nM BlaC, 0.2 nM CTX-M-14, and 0.2 or 0.25 nM TEM-1. Red lines represent experimental data, and black lines represent simulated data. Measurements were done in 100 mM NaPi buffer, pH 6.4, at 25 °C. WT – wild-type.

a

Tat-BlaC-pUK21

```
1       10         20         30         40         50         60
MANNDLFQAS RRRFLAQLGG LTVAGMLGPS LLTPRRATAA QADLADRFAE LERRYDARLG
                                                30         40
        70         80         90        100        110        120
VYVPATGTTA AIEYRADERF AFCSTFKAPL VAAVLHQNPL THLDKLITYT SDDIRSISPV
    50        60         70         80       90         100
       130        140        150        160        170        180
AQQHVQTGMT IGQLCDAAIR YSDGTAANLL LADLGGPGGG TAAFTGYLRS LGDTVSRLDA
 110        120        130        140            150        160
       190        200        210        220        230        240
EEPELNRDPP GDERDTTTPH AIALVLQQLV LGNALPPDKR ALLTDWMARN TTGAKRIRAG
     170        180        190        200        210        220
       250        260        270        280        290        300
FPADWKVIDK TGTGDYGRAN DIAVVWSPTG VPYVVAVMSD RAGGGYDAEP REALLAEAAT
     230       240        250      260         270        280
       310
CVAGVLALEH HHHHH
   290
```

Tat-CTX-M-14-pUK21

```
 10         20         30         40         50         60
MANNDLFQAS RRRFLAQLGG LTVAGMLGPS LLTPRRATAA QAQTSAVQQK LAALEKSSGG
                                                   30         40
        70         80         90        100        110        120
RLGVALIDTA DNTQVLYRGD ERFPMCSTSK VMAAAAVLKQ SETQKQLLNQ PVEIKPADLV
       50        60         70      80             90        100
       130        140        150        160        170        180
NYNPIAEKHV NGTMTLAELS AAALQYSDNT AMNKLIAQLG GPGGVTAFAR AIGDETFRLD
      110        120        130        140       150         160
       190        200        210        220        230        240
RTEPTLNTAI PGDPRDTTTP RAMAQTLRQL TLGHALGETQ RAQLVTWLKG NTTGAASIRA
      170        180        190        200        210        220
       250        260        270        280        290        300
GLPTSWTVGD KTGSGDYGTT NDIAVIWPQG RAPLVLVTYF TQPQQNAESR RDVLASAARI
      230       240        250       260        270        280
       310
IAEGLLEHHH HHH
    290
```

Tat-KPC-2-pUK21

```
 10         20         30         40         50         60
MANNDLFQAS RRRFLAQLGG LTVAGMLGPS LLTPRRATAA QALTNLVAEP FAKLEQDFGG
                                                   30        40
        70         80         90        100        110        120
SIGVYAMDTG SGATVSYRAE ERFPLCSSFK GFLAAAVLAR SQQQAGLLDT PIRYGKNALV
       50        60         70      80             90         100
       130        140        150        160        170        180
PWSPISEKYL TTGMTVAELS AAAVQYSDNA AANLLLKELG GPAGLTAFMR SIGDTTFRLD
      110        120        130        140       150         160
       190        200        210        220        230        240
RWELELNSAI PGDARDTSSP RAVTESLQKL TLGSALAAPQ RQQFVDWLKG NTTGNHRIRA
      170        180        190        200        210        220
       250        260        270        280        290        300
AVPADWAVGD KTGTCGVYGT ANDYAVVWPT GRAPIVLAVY TRAPNKDDKH SEAVIAAAAR
      230       240        250       260        270        280
       310
LALEGLGVNG QLEHHHHHH
     290
```

pelB-NmcA-pUK21

```
 10         20         30         40         50         60
MKYLLPTAAA GLLLLAAQPA MANTKGIDEI KNLETDFNGR IGVYALDTGS GKSFSYRANE
                            30        40          50        60
        70         80         90        100        110        120
RFPLCSSFKG FLAAAVLKGS QDNRLNLNQI VNYNTRSLEF HSPITTKYKD NGMSLGDMAA
     70      80             90         100       110        120
       130        140        150        160        170        180
AALQYSDNGA TNIILERYIG GPEGMTKFMR SIGDEDFRLD RWELDLNTAI PGDERDTSTP
     130        140         150        160        170        180
       190        200        210        220        230        240
AAVAKSLKTL ALGNILSEHE KETYQTWLKG NTTGAARIRA SVPSDWVVGD KTGSCGAYGT
      190        200        210        220        230        240
       250        260        270        280        290
ANDYAVVWPK NRAPLIISVY TTKNEKEAKH EDKVIAEASR IAIDNLKHHH HHH
      250       260        270        280        290
```

Sec-TEM-1-pUK21

```
 10         20         30         40         50         60
MSIQHFRVAL IPFFAAFCLP VFAHPETLVK VKDAEDQLGA RVGYIELDLN SGKILESFRP
                             30         40         50         60
        70         80         90        100        110        120
EERFPMMSTF KVLLCGAVLS RIDAGQEQLG RRIHYSQNDL VEYSPVTEKH LTDGMTVREL
       70      80             90         100       110        120
       130        140        150        160        170        180
CSAAITMSDN TAANLLLTTI GGPKELTAFL HNMGDHVTRL DRWEPELNEA IPNDERDTTM
       130        140        150        160        170        180
       190        200        210        220        230        240
PVAMATTLRK LLTGELLTLA SRQQLIDWME ADKVAGPLLR SALPAGWFIA DKSGAGERGS
       190        200        210        220        230       240
       250        260        270        280        290
RGIIAALGPD GKPSRIVVIY TTGSQATMDE RNRQIAEIGA SLIKHWHHHH HH
      250       260          270      280        290
```

b

BlaC-pET28a

```
1       10         20         30         40         50         60
MGSSHHHHHH SSGLVPRGSH MENLYFQSGG DLADRFAELE RRYDARLGVY VPATGTTAAI
                             ⇧     30         40         50
        70         80         90        100        110        120
EYRADERFAF CSTFKAPLVA AVLHQNPLTH LDKLITYTSD DIRSISPVAQ QHVQTGMTIG
 60         70         80      90         100        110        120
       130        140        150        160        170        180
QLCDAAIRYS DGTAANLLLA DLGGPGGGTA AFTGYLRSLG DTVSRLDAEE PELNRDPPGD
         130        140             150        160        170
       190        200        210        220        230        240
ERDTTTPHAI ALVLQQLVLG NALPPDKRAL LTDWMARNTT GAKRIRAGFP ADWKVIDKTG
   180        190        200        210        220        230
       250        260        270        280        290
TGDYGRANDI AVVWSPTGVP YVVAVMSDRA GGGYDAEPRE ALLAEAATCV AGVLA
  240        250      260          270        280        290
```

CTX-M-14-pET28a

```
 10         20         30         40         50         60
MGSSHHHHHH SSGLVPRGSH MENLYFQSGG QTSAVQQKLA ALEKSSGGRL GVALIDTADN
                             ⇧        30         40         50
        70         80         90        100        110        120
TQVLYRGDER FPMCSTSKVM AAAAVLKQSE TQKQLLNQPV EIKPADLVNY NPIAEKHVNG
    60         70      80           90            100       110
       130        140        150        160        170        180
TMTLAELSAA ALQYSDNTAM NKLIAQLGGP GGVTAFARAI GDETFRLDRT EPTLNTAIPG
    120        130        140        150        160        170
       190        200        210        220        230        240
DPRDTTTPRA MAQTLRQLTL GHALGETQRA QLVTWLKGNT TGAASIRAGL PTSWTVGDKT
    180        190        200        210        220        230
       250        260        270        280        290
GSGDYGTTND IAVIWPQGRA PLVLVTYFTQ PQQNAESRRD VLASAARIIA EGL
   240        250       260        270        280        290
```

TEM-1-pET28a

```
 10         20         30         40         50         60
MGSSHHHHHH SSGLVPRGSH MENLYFQSGG HPETLVKVKD AEDQLGARVG YIELDLNSGK
                            ⇧           30         40         50
        70         80         90        100        110        120
ILESFRPEER FPMMSTFKVL LCGAVLSRID AGQEQLGRRI HYSQNDLVEY SPVTEKHLTD
    60         70     80                90        100        110
       130        140        150        160        170        180
GMTVRELCSA AITMSDNTAA NLLLTTIGGP KELTAFLHNM GDHVTRLDRW EPELNEAIPN
    120        130        140        150        160        170
       190        200        210        220        230        240
DERDTTMPVA MATTLRKLLT GELLTLASRQ QLIDWMEADK VAGPLLRSAL PAGWFIADKS
    180        190        200        210        220        230
       250        260        270        280        290
GAGERGSRGI IAALGPDGKP SRIVVIYTTG SQATMDERNR QIAEIGASLI KHW
   240        250       260            270     280        290
```

Figure S3. The amino acid sequences of all β-lactamases used in this study. (a) The pUK21 construct used in fitness experiments and minimum inhibitory concentration (MIC) determinations. The N-terminal translocation signal peptide (Tat/pelB/Sec) is indicated in red, and the C-terminal 6xHis tag with two additional residues (LE) is indicated in blue. (b) The pET28a construct used for the production of recombinant BlaC, CTX-M-14, and TEM-1 proteins, and for *in vitro* characterization. The N-terminal, cleavable 6xHis tag on the N-terminal is shown in blue, the TEV cleavage sequence in brown, with the arrow denoting the position where the cleavage occurs. Note that two additional Gly residues were inserted to enhance the efficiency of TEV cleavage. The upper numbering corresponds to the actual sequence, and the lower to the Ambler notation [1].

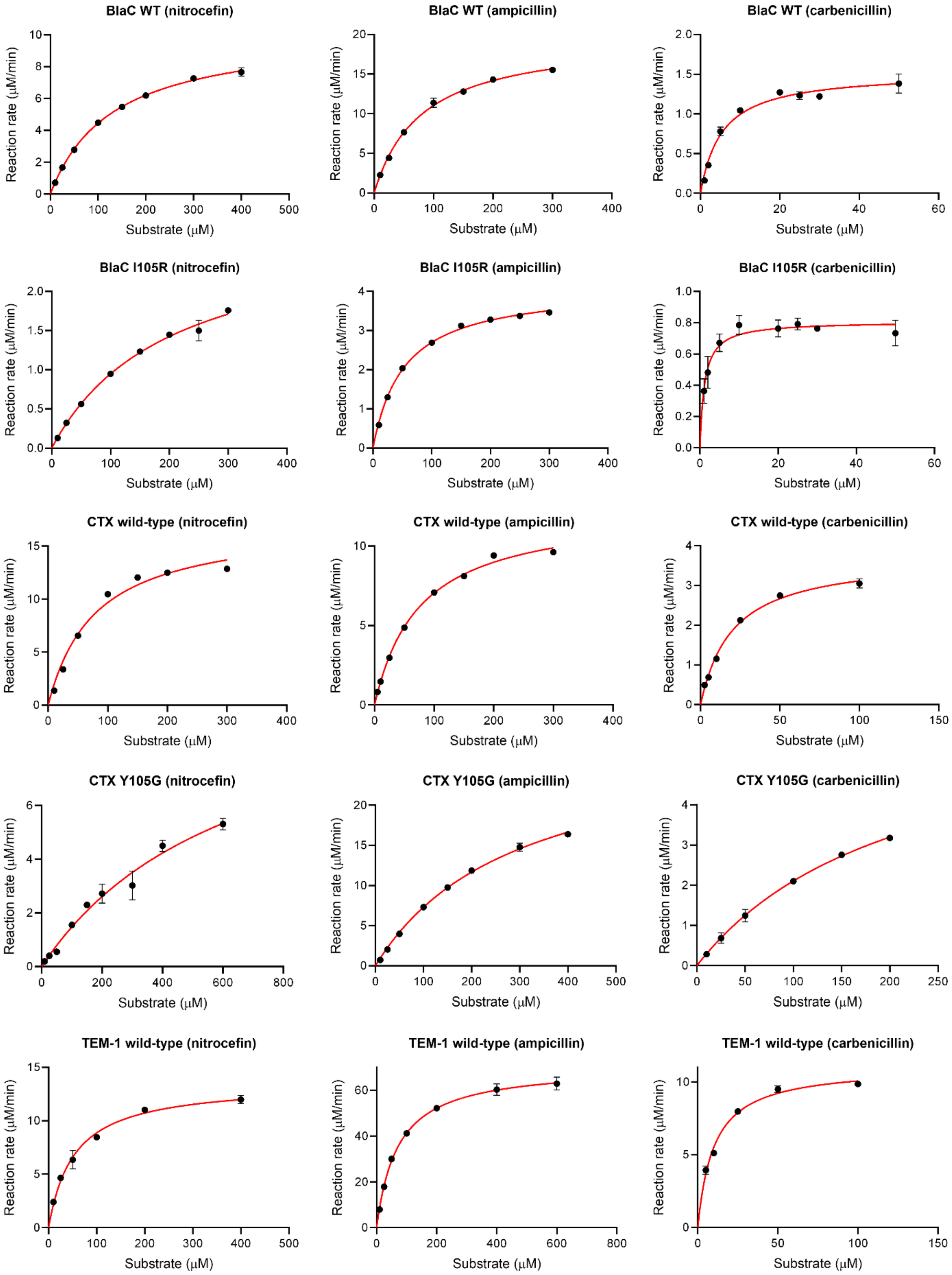
BlaC WT (nitrocefin)
BlaC WT (ampicillin)
BlaC WT (carbenicillin)
BlaC I105R (nitrocefin)
BlaC I105R (ampicillin)
BlaC I105R (carbenicillin)
CTX wild-type (nitrocefin)
CTX wild-type (ampicillin)
CTX wild-type (carbenicillin)
CTX Y105G (nitrocefin)
CTX Y105G (ampicillin)
CTX Y105G (carbenicillin)
TEM-1 wild-type (nitrocefin)
TEM-1 wild-type (ampicillin)
TEM-1 wild-type (carbenicillin)
Reaction rate (μM/min)
Substrate (μM)

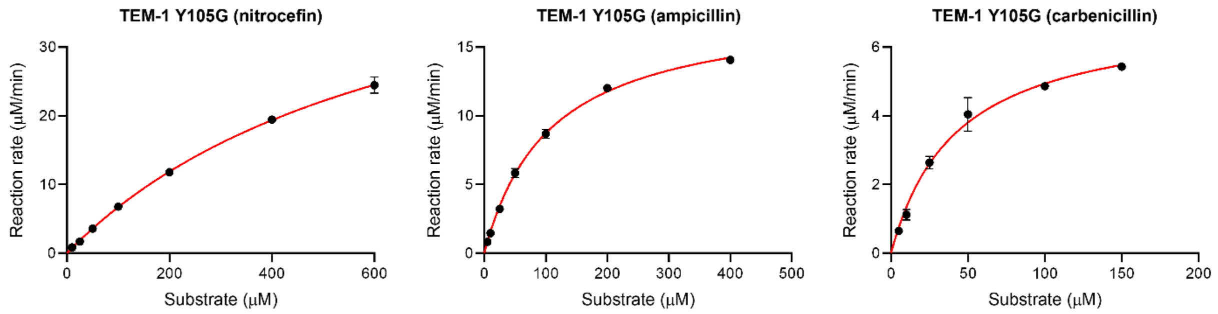

Figure S4. Michaelis-Menten plots of all BlaC, CTX-M-14, and TEM-1 variants characterized in this study using nitrocefin, ampicillin, and carbenicillin as substrates. The red line represents a non-linear or linear fit to the experimental data points. Errors represent one standard deviation of the triplicate measurements.

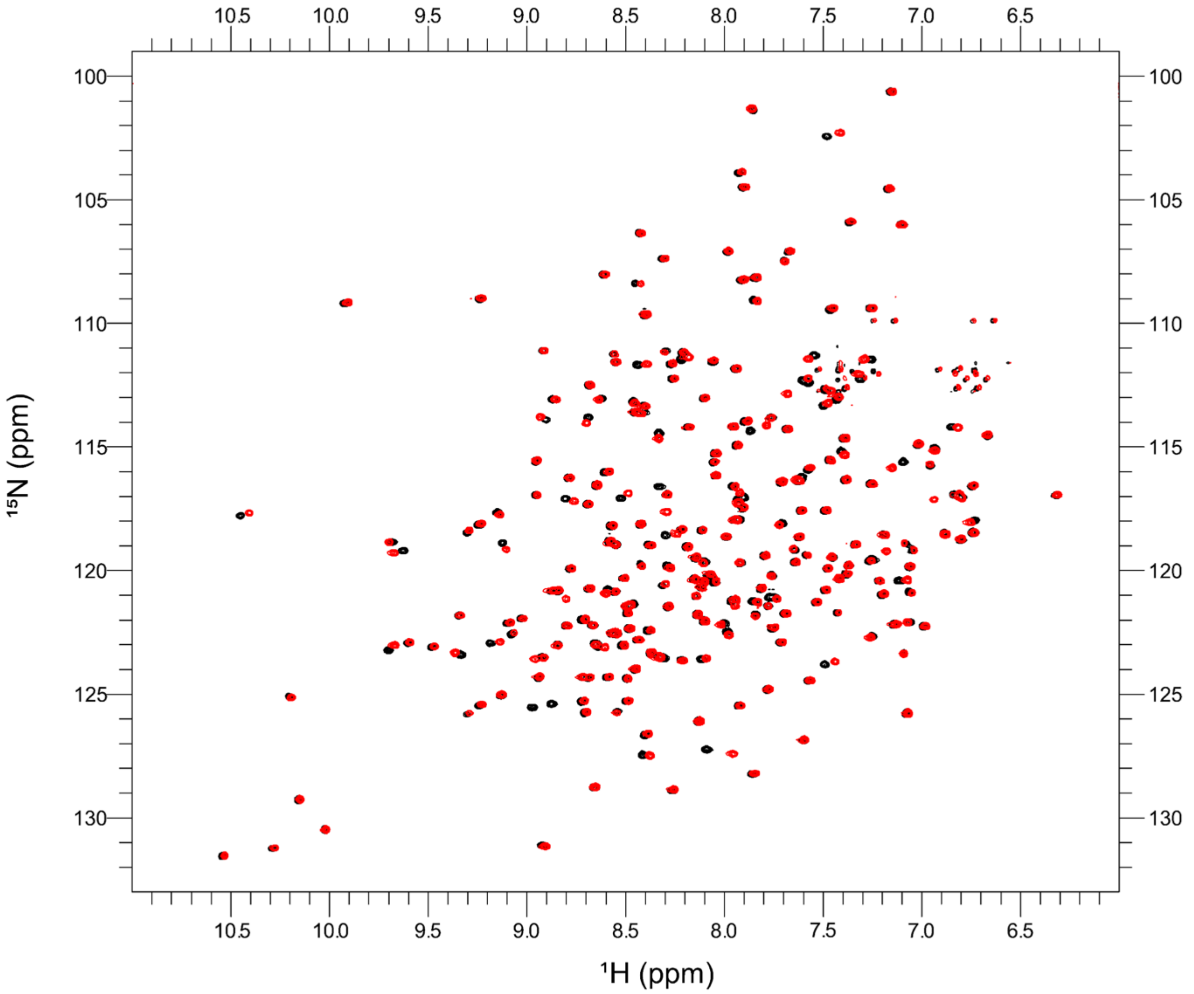

Figure S5. Overlay of ^{1}H-^{15}N TROSY-HSQC spectra for BlaC I105R (red) and wild-type (black).

Octave script for the simulation of inhibition curves

```
#! C:\Octave\Octave-6.2.0\share\octave\site\m\startup\octaverc
# Model:
# S(1) + E(2) k2<Ki>k1 SE(3) >k3 PSE(4) >k4 PS(5) + E(2)
# I(6) + E(2) k6<K2i>k5 IE(7) >k7 PIE(8) >k8 PI(9) + E(2)

clear all

# Load data and adjust
data = load ("I105R_CA_inhibition.csv");
time = data(:,1);
conc = load ("I105R_CA_concentrations.csv"); #conc of inhibitor

# Set user variables
plcon = 3; #number in the substrate list for which all elements are plotted
enz = 0.002; #total enzyme conc in micromolar

# Set variables
num1 = numel(conc);
num2 = numel(time);
num3 = columns(data);
prod = data(:,2:num3);
res = zeros(num2,num1);

for i = 1:num1

con = conc(i);

# Define rates and differential equations
function xdot = f (x,t)
  Kd1 = 150;
  k1 = 0.18;
  k2 = Kd1*k1;
  k3 = 2000;
  k4 = 57;
  Kd2 = 110;
  k5 = 1;
  k6 = Kd2*k5;
  k7 = 0.046;
  k8 = 0.002;
  xdot = zeros (9,1);
  xdot(1) = -k1*x(1)*x(2)+k2*x(3);
  xdot(2) = -k1*x(1)*x(2)+k2*x(3)+k4*x(4)-k5*x(6)*x(2)+k6*x(7)+ k8*x(8);
  xdot(3) = k1*x(1)*x(2)-(k2+k3)*x(3);
  xdot(4) = k3*x(3)-k4*x(4);
  xdot(5) = k4*x(4);
  xdot(6) = -k5*x(6)*x(2)+k6*x(7);
  xdot(7) = k5*x(6)*x(2)-(k6+k7)*x(7);
  xdot(8) = k7*x(7)-k8*x(8);
  xdot(9) = k8*x(8);

endfunction;
x0 = [125; 0.002; 0; 0; 0; con; 0; 0; 0];
t = logspace(-3,6,1000);           #adjust to see entire range
y1 = lsode ("f", x0, t);
y2 = lsode ("f", x0, time);
res(:,i) = y2(:,5);
```

```
if (i == plcon)
  plc = y1;
endif

endfor

# Plot the results

# Normal plot of data and simulated product curves
figure('Position',[300,250,1000,500]);
#subplot (1, 2, 1);
plot (time, prod, "-r","linewidth", 1, time, res,"-k", "linewidth", 1);
axis([0, 600, 0, 20]);
set(gca, "linewidth", 2, "fontsize", 11)
xlabel ("Time (s)", "fontsize", 16);
ylabel ("Hydrolyzed nitrocefin (μM)", "fontsize", 16);

# Log plot of all elements for the last concentration
#subplot (1, 2, 2);
#plot (log10(t),log10(plc),"linewidth", 1);
#axis([-3,6,-6, 3.2]);
#set(gca, "linewidth", 2, "fontsize", 12);
#xlabel ("log(10) time (s)");
#ylabel ("log(10) concentration (uM)");
#legend ("S", "E", "SE", "PSE", "PS", "I", "IE", "PIE", "PI", "location", "west");

print -djpg I105R_CA_inhibition_curves.jpg;
```

SUPPORTING REFERENCES

1. Ambler, R. P. *et al.* A standard numbering scheme for the class A β-lactamases. *Biochem J* **276**, 269–270 (1991).
